# Longitudinal prediction of DNA methylation to forecast epigenetic outcomes

**DOI:** 10.1016/j.ebiom.2025.105709

**Published:** 2025-04-22

**Authors:** Arthur Leroy, Ai Ling Teh, Frank Dondelinger, Mauricio A. Alvarez, Dennis Wang

**Affiliations:** a Department of Computer Science, The University of Manchester, Manchester, United Kingdom; b Department of Computer Science, The University of Sheffield, Sheffield, United Kingdom; c Institute for Human Development and Potential (IHDP), Agency for Science, Technology and Research (A∗STAR), Singapore, Republic of Singapore; d Bioinformatics Institute (BII), Agency for Science, Technology and Research (A∗STAR), Singapore, Republic of Singapore; e Lancaster Medical School, Lancaster University, Lancaster, United Kingdom; f National Heart and Lung Institute, Imperial College London, London, United Kingdom

**Keywords:** DNA methylation, Epigenetic age, Longitudinal data, Machine learning

## Abstract

**Background:**

Epigenetic changes in early life play an important role in the development of health conditions in children. Longitudinally measuring and forecasting changes in DNA methylation can reveal patterns of ageing and disease progression, but biosamples may not always be available.

**Methods:**

We introduce a probabilistic machine learning framework based on multi-mean Gaussian processes, accounting for individual and gene correlations across time to forecast the methylation status of an individual into the future. Predicted methylation values were used to compute future epigenetic age and compared to chronological age.

**Findings:**

We show that this method can simultaneously predict methylation status at multiple genomic sites in children (age 5–7) using methylation data from earlier ages (0–4). Less than 10% difference between observed and predicted methylation values is found in approximately 95% of methylation sites. We show that predicted methylation profiles can be used to estimate other molecular phenotypes, such as epigenetic age, at any timepoint and enable association tests with health outcomes measured at the same timepoint.

**Interpretation:**

Limited longitudinal profiling of DNA methylation coupled with machine learning enables forecasting of epigenetic ageing and future health outcomes.

**Funding:**

10.13039/100010269Wellcome Trust, 10.13039/501100001381Singapore National Research Foundation (NRF), Singapore 10.13039/501100001349National Medical Research Council (NMRC), Agency for Science, Technology and Research (A∗STAR), UK 10.13039/501100000691Academy of Medical Sciences and the UK 10.13039/501100000266Engineering and Physical Sciences Research Council (EPSRC).

**Research in context**

**Evidence before this study**

Epigenetic changes and DNA methylation reflect the cumulative effects of genetic and environmental exposures. These changes are crucial in understanding long-term health outcomes, ageing, and disease mechanisms. Previous studies primarily relied on cross-sectional data, which limited the ability to capture dynamic epigenetic modifications over time. Moreover, current methods examine methylation sites one at a time and within a single time point and lack the capability to study methylomes at timepoints when data were not collected. Machine learning has demonstrated the ability to model covariation between multiple dimensions and forecast future events. It has yet to be seen whether this can simultaneously model the covariation between methylation sites, individuals and across timepoints.

**Added value of this study**

This study shows that multi-mean Gaussian processes trained on historic measures of DNA methylation in a cohort of children can forecast future methylomes of individuals when it is not possible to collect biosamples to measure methylation directly. We demonstrate and provide one of the largest datasets to predict future methylation profiles, estimate epigenetic ages and test for association with health outcomes in children.

**Implications of all the available evidence**

This approach addresses the challenge of missing biosamples during critical health outcome assessments, allowing for predicting molecular biomarkers even when direct measurements are unavailable. It extends the scope of traditional cross-sectional analyses, enabling researchers to forecast epigenetic outcomes and their impact on health, thus advancing personalised interventions.

## Introduction

Longitudinal molecular profiling is pivotal to advancing ageing research by providing critical insights into development, growth and, ultimately, frailty. By integrating a multi-omics approach with clinical data, such studies capture the dynamic nature of diseases and reveal the underlying molecular signatures of ageing and disease processes. Previous studies have demonstrated the power of longitudinal molecular profiling in tracking tumour evolution and identifying therapeutic targets in patients with colorectal cancer.^1^^,^^2^ More recent investigation on population cohorts showcased how longitudinal molecular profiling aided in predicting treatment outcomes and tailoring interventions for patients with chronic diseases, such as coronary heart disease.^3^^,^^4^ However, when health outcomes are recorded, there may be a lack of biosamples available to measure the molecular changes occurring at that time. The insufficient sampling of readouts may result in underpowered studies.^5^

A particular type of molecular profile, DNA methylation, can reflect the cumulative effects of both genetic and environmental exposures, making them ideal candidates for studying long-term health outcomes. Cross-sectional studies often only consider measures on a single time point, overlooking potential longitudinal information that might be valuable. By assessing DNA methylation at multiple time points, researchers can investigate how epigenetic modifications dynamically respond to environmental stimuli, ageing, and health status progression. Epigenetic age has been widely studied in the field these days. Researchers can estimate the epigenetic age as a function of methylation values from different signature CpGs set.^6^, ^7^, ^8^, ^9^ Moreover, DNA methylation alterations have been associated with a wide range of diseases, including cardiovascular disease,^4^ diabetes,^10^ psychiatric conditions,^11^^,^^12^ and cancer.^13^ Longitudinal studies incorporating DNA methylation profiles can provide insights into biological ageing processes and disease mechanisms, however, this information needs to be measured repeatedly to capture the most important changes.

To date, there have only been algorithms to predict additional methylation sites at the same time point as the available data^14^^,^^15^ for training. Clinicians and researchers aiming to examine methylation status at other time points when health outcomes are measured would not be able to do so if biosamples were not collected. Here, we aspire to extend the traditional cross-sectional DNA methylation analyses with a broader longitudinal approach to modelling DNA methylation across the early lifespan of children in a multi-ethnic population cohort. We demonstrate that DNA methylation levels for any set of CpG sites can be predicted using the methylation patterns measured years earlier. We showed this for CpGs of different epigenetic clocks and then correlate routine health measures with epigenetic age estimates from predicted methylation profiles.

## Methods

This section describes the studied cohort, the variables of interest, and how the data was collected. We also describe step-by-step, including technical details, how to derive probabilistic epigenetic age predictions from methylation time series.

### Description of the child cohort

The subjects in this analysis are participants of the Growing Up in Singapore Towards healthy Outcomes (GUSTO) birth cohort.^16^ Briefly, this multi-ethnic study contains about 1400 mother–child pairs with dense phenotypes. Mothers were recruited at two participating hospitals during the first trimester of pregnancy and followed over time through regular clinic or home visits. The children of these women are also followed over time after birth. To conduct this analysis, we selected 110 training participants for whom we had access to longitudinal methylation data profiles at 3, 9, 48 and 72 months. We then validated our model using another set of 188 testing participants with methylation data available at the same time points mentioned. The basic demographics of these subjects are shown in Table 1. This subset of samples is representative of the cohort with a similar distribution of ethnic groups^16^ as well as male and female participants. The data are collected at 4 different timepoints: 3, 9, 48, and 72 months, however, the exact time of data collection could differ from one individual to another. All computations were performed using the exact date of data collection to account for the possible influence of time lags in measurements.

**Table 1:** Demographic Information of training and testing samples.

Variables	Training: timepoints				Testing: timepoints			
	3 months	9 months	48 months	72 months	3 months	9 months	48 months	72 months
	N (%)/Mean (sd)				N (%)/Mean (sd)			
Ethnicity								
Chinese	61 (55.5%)				114 (60.6%)			
Malay	30 (27.3%)				41 (21.8%)			
Indian	19 (17.3%)				33 (17.6%)			
Sex								
Male	53 (48.2%)				93 (49.5%)			
Gestational age (weeks)	38.8 (1.1)				38.7 (1.3)			
Birthweight (kg)	3.1 (0.4)				3.1 (0.5)			
Age at collection (years)	0.26 (0.02)	0.76 (0.02)	4.05 (0.09)	6.05 (0.10)	0.25 (0.02)	0.76 (0.03)	4.08 (0.09)	6.06 (0.09)
Weight (kg)	6.1 (0.8)	8.6 (1.0)	16.3 (3.4)	20.8 (4.9)	6.1 (0.8)	8.5 (1.1)	16.5 (3.0)	20.9 (4.3)
Length/Height (cm)	60.9 (2.4)	71.5 (2.9)	101.7 (4.8)	114.8 (5.5)	60.9 (2.5)	71.3 (2.9)	102.3 (4.3)	115.4 (5.0)
Head circumference (cm)	39.9 (1.2)	44.5 (1.4)	49.8 (1.4)	50.9 (1.5)	39.9 (1.4)	44.3 (1.4)	49.8 (1.5)	51.0 (1.5)
Systolic blood pressure	–	–	–	102.0 (9.0)	–	–	–	100.2 (7.8)
Diastolic blood pressure	–	–	–	60.2 (5.8)	–	–	–	59.4 (5.6)

### DNA methylation data

Buccal swab samples were collected from the participants by research staff using Isohelix swabs (SK-2S) during clinic visits and stored at −80 °C freezer. DNA was then extracted from buccal swabs using IsoHelix DNA Xtreme Kit (Cat. No: XME-5/50) (0–4 years samples) and Qiasymphony DSP DNA Midi Kit (Cat No: 937255) from Qiagen Singapore Pte Ltd (6 years samples) following the standard protocol recommended by the company.

Extracted DNA samples were sent for DNA methylation profiling using the Infinium MethylationEPIC BeadChip array (EPIC850k). DNA methylation profiling was done as per Illumina standard protocol. Data quality control (QC) and preprocessing were performed in R (version 4.0). The raw .idat files were read and processed using minfi package (version 1.42.0).^17^ The methylation value for a CpG was denoted “NA” when the detection p-value >0.01 or NBeads <3. A CpG was removed from the analysis if more than 5% of the samples failed the quality control (QC) criteria.

### Clinical variable collection

Clinical variables such as diastolic blood pressure and systolic blood pressure were collected during visits to the National University Hospital (NUH) and KK Women's and Children's Hospital in Singapore. Anthropometric data such as weight and height were also measured during the annual follow-ups. Physical activity data of the children were collected using a wearable accelerator as previously reported.^18^

### Longitudinal modelling

In order to model the methylation values over time for each CpG and individual as continuous functions (i.e. time series), we proposed a framework based on Gaussian processes, which can be considered as an extension of the algorithm presented in Leroy et al.^19^ We called this framework *multi-mean Gaussian processes* to emphasise the strategy leveraging multiple latent mean processes to provide adaptive predictions in cases of a large number of time series presenting multiple sources of correlation.

In the present study, we can define two separate sources of correlations in our data: the individuals and the CpGs (as we can expect time series coming from the same individual, or CpG, to present related patterns). Let us denote *y*^*j*^(*t*) the DNA methylation value associated with the *i*-th individual, the *j*-th CpG, observed at a time *t*. From a mathematical point of view, the proposed multi-mean Gaussian processes (GP) model can be expressed as follows:

(1) $$y_{i}^{j}\left(t\right)=\mu_{0}\left(t\right)+f_{i}\left(t\right)+g^{j}\left(t\right)\;+\;\varepsilon_{i}^{j}\left(t\right),\forall t\;\epsilon \;Τ,\forall i\in I,\forall j\in J$$

where $\mu_{0}\left(\cdot \right)∼GP\;\left(m_{0}\left(\cdot \right),k_{\theta_{0}}\left(\cdot ,\cdot \right)\right)$ is a mean common process, while $f_{i}\left(\cdot \right)∼GP\;\left(0,k_{\theta_{i}}\left(\cdot ,\cdot \right)\right)$ represents an individual-specific process, and $g^{j}\left(\cdot \right)∼GP\;\left(0,k_{\theta^{j}}\left(\cdot ,\cdot \right)\right)$ a CpG-specific process. Moreover, the error term is supposed to be $\epsilon_{i}^{j}\left(\cdot \right)∼GP\left(0,\sigma_{i}^{j}\;I_{d}\right)$. All covariance kernels are assumed to be *squared exponentiated* in the following experiments.

**Fig. 1:**
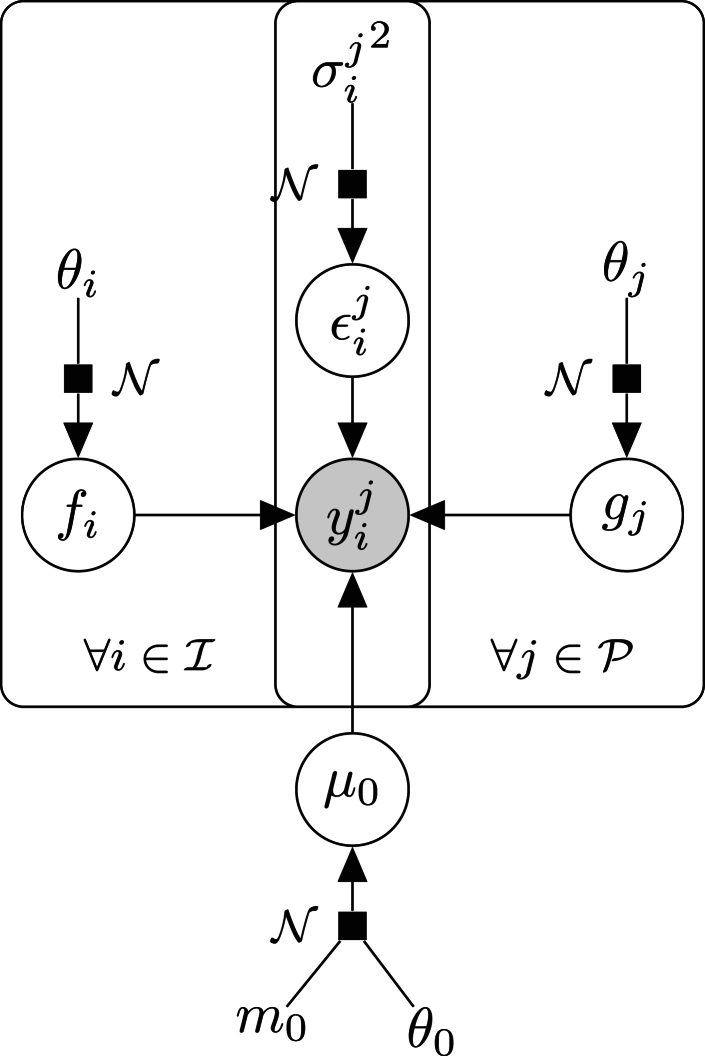
**Graphical model of dependencies between variables in the multi-mean Gaussian processes model**.

Intuitively, this means that any time series $y_{i}^{j}$ is assumed to be the sum of a common mean trend (μ_0_), a perturbation coming from the individual (*f*_*i*_), and another perturbation specific to the CpG (*g*_*j*_). For readers familiar with mixed models in statistics, one can retrieve ideas roughly similar to the notions of fixed and random effects, although we are here working with continuous functions instead of vectors. For the sake of clarity, the graphical model, illustrating this data generative process and the modelling assumptions, is provided in Fig. 1.

### Methylation prediction

**Fig. 2:**
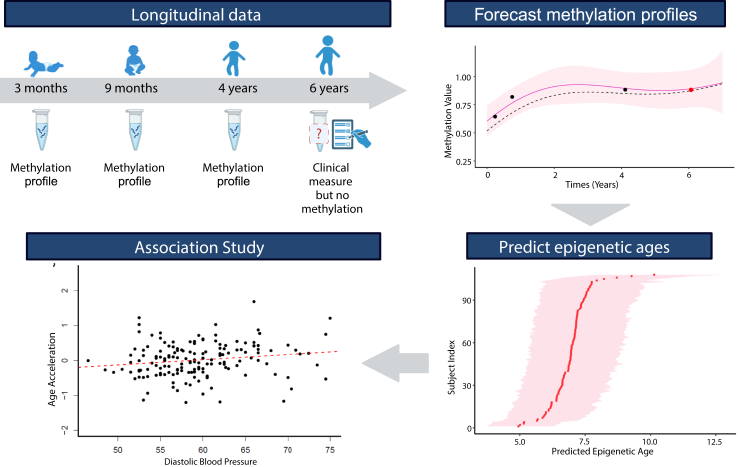
**Schematic overview of predicting methylation profiles and its application**. In human studies, there are times when molecular measures, such as DNA methylation values, were not collected when there were clinical measures. For instance, there could be blood pressure at year 6 but no methylation data. The missing methylation data at CpG sites needed for analysis can be predicted using longitudinal data collected at earlier time points using multi-mean Gaussian processes. Phenotypes, such as epigenetic age can then be computed from the predicted methylation values and used to study associations with clinical traits (i.e. diastolic blood pressure).

Setting aside mathematical sophistication, it is possible to leverage an Expectation-Maximisation (EM) algorithm (as proposed in Leroy et al.^19^) to estimate both the model parameters and the mean process from data. More specifically, the key idea of the present framework consists in computing multiple mean processes. Each mean process is associated with a specific CpG by computing a posterior distribution conditioned over an adequate subset of data through Bayes’ law. This method allows us to derive CpG-specific mean processes, which are particularly relevant for predicting future values of the time series. One can think of those processes as averaged curves over all individuals, along with their associated uncertainty quantification. An example of CpG-specific mean processes is displayed in Fig. 2 to illustrate the ability of multi-mean GPs to recover an adaptive trend from each subset of data accurately. These mean processes are of utmost importance when it comes to predicting unobserved values for a specific individual. Whether the goal consists in predicting missing data or (possibly long-term) forecasting from a handful of points, those mean processes provide a powerful way to implicitly transfer knowledge across individuals to improve performance in these tasks. The current implementation of the framework, based on the *R* package MagmaClustR,^20^ is provided in the following repository: https://github.com/ArthurLeroy/MultiMeanGP. As a probabilistic non-parametric machine learning method, Gaussian processes are known to be reasonably robust to overfitting, particularly in such multi-task contexts where hyperparameters are trained and shared across several individuals. The marginal likelihood that is optimised during the training step involves a trace of covariances term, which acts as a natural regulariser to mitigate overfitting pitfalls. Moreover, the uncertainty quantification of GPs automatically adapts according to the sample size and dispersion of training data. We validated that the Credible Intervals (CI) that we computed theoretically were also well-calibrated empirically through a dedicated metric of uncertainty coverage detailed in Method section.

We considered the time series of methylation profiles for 110 training individuals observed at the CpG sites of skin&blood clock and PedBE clock. The original epigenetic clock contains 391 and 94 CpGs respectively. We have removed probes that failed QC in our data and left with 368 (skin&blood clock) and 91 (PedBE clock) CpG sites, respectively. We have specifically chosen CpGs in PedBE and skin&blood clocks because they are the best correlated with epigenetic age clocks for our data that was derived using children buccal swab samples (Table 2). Each time series is specific to an individual-CpG couple and consists of measurements collected at 4 timestamps (3, 9, 48 and 72 months). The objective resides in predicting methylation values at 72 months for any of the 188 testing individuals who were partially observed (i.e. with observed data only at 3, 9 and 48 months or any subset of these). From a mathematical point of view, this problem is far from trivial as such long-term forecasting tasks (a 2-year gap between the last observation and the target value) from only 3 data points, and will generally lead to uncertain results. However, leveraging the mean processes trained with the multi-mean GPs algorithm can greatly enhance performance by sharing information and uncertainty quantification across individuals. For two different individuals, the prediction of multi-mean GPs benefits both from the mean process’ ability to recover the long-term trend of this example CpG, as well as the specific pattern coming from the study participant. The methylation forecasting step ends when we obtain a predicted value at 72 months for all 188 individuals and all CpGs of interest. Then, from these predicted methylation values, we can compute the epigenetic age using existing epigenetic clocks.

**Table 2:** Correlation of different epigenetic age clocks with chronological age.

Epigenetic age clocks	Correlation
Horvath	−0.0646
Levine	0.0159
skinHorvath	0.1666
Wu	0.0395
PedBE	0.1005

### Estimating epigenetic age

As previously mentioned, various methods exist nowadays to compute epigenetic age from an adequate CpG signature. In the present paper, we took advantage of two well-known epigenetic clocks, the Horvath skin&blood^21^ and the PedBE^9^ clocks. Both methods leverage the same functional form to express the relationship between epigenetic age and methylation values through the following equation (see Horvath,^6^ Additional file 2):

(2) $$age=21\times \exp\left(c^{T}x\right)\;-1$$

where x represents the vector of methylation values for each CpG and c the vector of associated coefficients. The above expression is derived from the original formulation in Horvath's article, which proposed to transform the age before applying elastic net regression through the following function: $f\left(age\right)=\log\left(\frac{age\;+\;1}{adult.age\;+\;1}\right)$. This stands for age≤adult.age, with adult.age=20 by convention in Horvath's clock. While the formula is similar for both clocks, the subset of CpGs involved in the computation differs. Therefore, x and c are 368-dimensional vectors in the Horvath skin&blood clock and 91-dimensional vectors in the PedBE clock. The clock corresponds to eq. (2), along with a vector of coefficients c, which differs depending on the method used. The vector of methylation values x is generally observed, thus allowing direct computation of epigenetic age.

In our study, we used the predicted methylation values as a surrogate to the true observations x. As our predictions are defined as Gaussian distributions, we cannot extract a single vector x without losing information about uncertainty quantification. In order to propagate the probabilistic nature of our forecasting through eq. (2), we have generated a large number of samples (10,000 samples for each clock) from the predictive GP distributions associated with each CpG. Hence, each sample corresponded to a single vector x for which estimating the epigenetic age through eq. (2). Finally, the resulting set of 10,000 epigenetic age estimations provided an empirical distribution accounting for the full uncertainty in our predictions. Although we proposed 10,000 samples as a sufficient number to obtain accurate empirical estimates, our implementation allows for an arbitrary increase in this number to achieve results that closely match the theoretical distribution. In order to evaluate the accuracy of our predictions, we compared them to the actual epigenetic age (computed from the observed methylation values at 72 months). We have also compared using Pearson correlation the epigenetic age computed using the predicted methylation value to the true age of data collection for all individuals. The computational cost of the prediction step in GP-based models is marginal compared with training. For each individual-CpG time series, the posterior distribution (i.e. the full prediction curve with its associated credible interval) can be computed in less than a second. For reference, the overall evaluation of the 69,184 individual-CpG predictions used by the Horvath skin&blood clock has been completed in less than 2 h on a laptop (HP EliteBook 840) with a 12-core CPU (Intel Core Ultra 7 165U) and 32 GB of RAM. The propagation of uncertainty when computing epigenetic age, by drawing 10,000 samples from the posterior distribution for each individual-CpG time series, was achieved in about 10 s on the same laptop.

### Evaluation metrics

Let us now denote *N* as the number of individuals, *T*_*i*_ the number of time points observed for the *i*-th individual, and *y*_*obs*_ and *y*_*pred*_ represent the vectors of observed and predicted methylation values, respectively. We define the Root Mean Squared Error (RMSE) in the subsequent experiments as follows:

(3) $$\sqrt{\frac{1}{N\times T_{i}}\sum_{i=1}^{N}\sum_{t=1}^{T_{i}}(y_{i}^{obs}{\left(t\right)-y_{i}^{pred}\left(t\right))}^{2}}$$

Moreover, an additional measure of uncertainty quantification is used to evaluate whether the observations belong to the predicted credible interval as expected. Namely, the *CI*_*95*_ Coverage (*CIC95*) is defined as:

(4) $$100\times \frac{1}{N}\sum_{i=1}^{N}l_{\left\{y_{i}^{obs}\epsilon \;{CI}_{95}\right\}}$$

where *CI*_*9*5_ represents the 95% credible interval computed from the predictive Gaussian distribution. When interpreting this metric, the closer to the theoretical value of 95%, the better.

### Baseline methods for comparison

To evaluate the mean predictive performances, we compared our results to several natural baselines. First, we defined the *individual mean*, which is the average of methylation values at 3, 9, and 48 months as an estimation of the individual typical values. Conversely, we also proposed the *CpG-mean* as another estimator, computed as the average of methylation values at 6 years from all other individuals. Another naive approach, which we called 48-month value, consists of using the observed value at 48 months as a predictor of methylation at 72 months. Finally, we proposed a comparison with mixed linear models.

### Statistics

All statistical analyses were performed in RStudio (version 2022.12.0 Build 353).^22^ Linear regression was performed to study the association between age acceleration (AA) and clinical relevance variables. We performed the linear regression using both AA computed from observed and predicted methylation values to access the application of the method. We computed Pearson and Spearman correlation coefficients and reported them in associated figures. Statistical analyses involving clinical variables only included cohort participants with measured clinical variables.

### Ethics

Ethics approval for the GUSTO cohort was obtained from the Centralised Institutional Review Board of SingHealth (2018/2767/D) and the Domain Specific Review Board of Singapore National Healthcare Group (D/2009/00021). Parents of all participating parents signed written informed consent for themselves and on behalf of their offspring.

### Role of funders

The funding agencies of this study played no role in study design, data collection, data analysis, interpretation, or writing of this manuscript.

## Results

To demonstrate our framework and evaluate its performance, the present section illustrates the forecasting of methylation profiles and their utilisation in predicting the epigenetic age. The omics data and phenotypes presented throughout is a subset of subjects of the GUSTO birth cohort.^16^ From longitudinal omics data, through the multi-mean GP algorithm to forecast methylation values and obtain epigenetic age predictions. Researchers can utilise this approach to explore the impact of DNA methylation at future time points and assess its correlation with diverse health outcomes (Fig. 2).

### Modelling of longitudinal methylation with uncertainty

To study multiple DNA methylation time series simultaneously and forecast their future values, we developed a tailored machine-learning framework, based on recent developments in multi-task GPs.^19^ The pivotal advancement comes from sharing information across all observed individuals and CpG sites by leveraging multiple adaptive mean processes, hence the name multi-mean GPs.

**Fig. 3:**
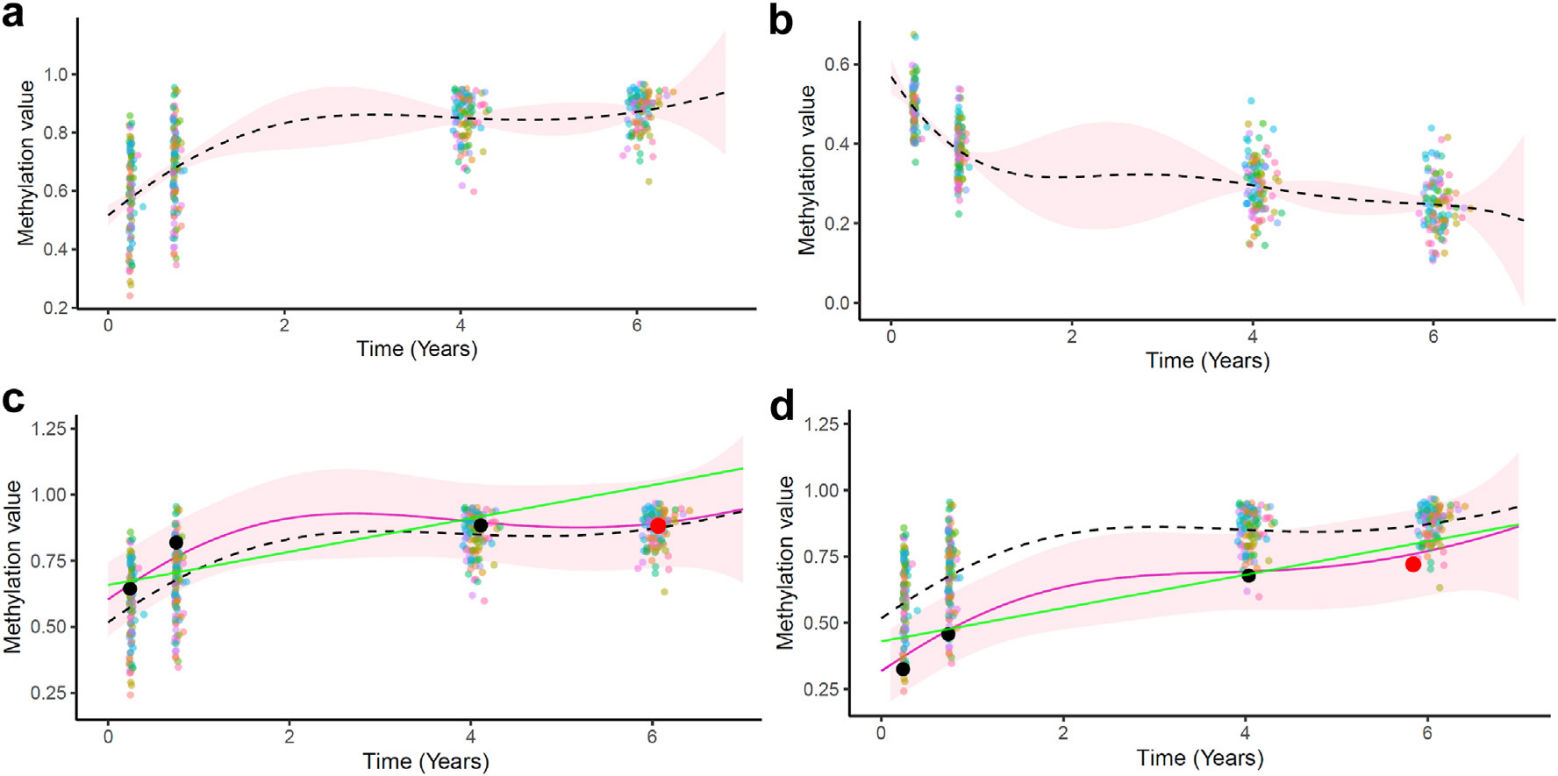
**CpG-specific mean processes and individual-specific predictions**. CpG-specific mean processes (dashed line) with associated 95% credible interval (pink band) differs between two illustrative CpGs (a, b). Using the CpG from panel a, Multi-mean GPs prediction curve (pink) with associated 95% credible intervals (pink band) for two illustrative individuals are different (c, d). The dashed line represents the mean curve from the CpG-specific mean process. The green line represents the fit from a mixed linear model where there is no learning between CpGs and individuals. Observed points are coloured in black, while the testing point is in red. Background points correspond to the training observations coloured by individuals.

For each CpG, the algorithm recovers the average trend of methylation values changes over time (Fig. 3a and b). The mean estimates and their associated 95% credible interval are estimated from data of each CpG across different time points. The two examples show that the longitudinal trend and associated uncertainty of mean processes can largely differ from one CpG to another. Such adaptive behaviour is essential when it comes to predicting unobserved data points accurately from a handful of observed points. We notice that the estimated uncertainty increases in regions lacking observations (95% credible interval widens from 0.02 at 9 months to 0.2 around 2 years). This is a characteristic feature inherent in GP-based methodologies that align with our expectations. Conversely, the CI around the mean curve becomes narrow near locations with dense data points. This is an anticipated outcome, as the abundance of information in those areas allows for more assured and precise estimates.

Although surprising at first sight, it is reasonable that the uncertainty for one individual does not cover the full range of observed methylation values in the cohort, which can be widespread across individuals (Fig. 3a). Although there is a high variance in methylation values from one individual to another, the estimation of their shared mean is robust or have low uncertainty when the number of data points increases.

### Multi-mean GPs models can predict methylation status 2 years into the future

**Fig. 4:**
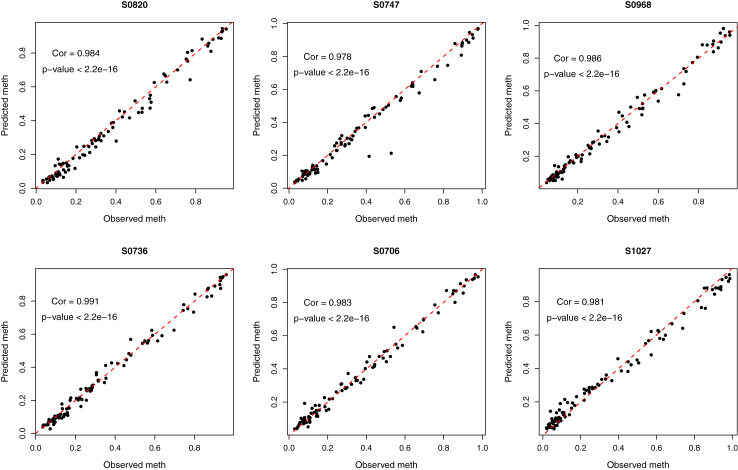
**Predicted methylation values versus measured methylation values for the 91 CpGs in PedBE̵7;s clock**. Example of predicted methylation values (y-axis) plotted against observed methylation values (x-axis) using 6 subjects from the testing set. Each dot represents a CpG in the PedBE epigenetic clock signature. The red dotted line represents the x = y line, and Spearman correlations are reported.

Once mean processes have been computed for all CpGs involved, we aimed to forecast CpG status for all individuals at specific timepoints. We predicted methylation status at the 6-year time point based on the observations at 3, 9 and 48 months. In Fig. 3, we provide an illustration of the results obtained from the prediction step of the multi-mean GPs algorithm. By integrating out the CpG-specific mean processes, one can compute a Gaussian posterior distribution for any individual time series in closed form (mathematical details can be found in Leroy et al., Proposition 5). As illustrated with the example CpG (Fig. 3a), we show how the mean process acts as a common trend across all individuals, representing the general evolution pattern for this CpG (Fig. 3c and d). The prediction for each individual deviates from its prior mean by adapting its trajectory to the observed data points from the individual of interest, which provides individual-specific information to enhance the accuracy of the predicted data point. Without multi-mean GP, every subject will get the same predicted value at age 6 regardless of the previous data (information). Expanding the evaluation from single CpG, let us evaluate the proposed method's performances over 188 testing individuals and their corresponding methylation profiles of 368 and 91 CpGs for skin&blood^21^ and PedBE^9^ epigenetic clock datasets, respectively. As multi-mean GPs provide a full probability distribution for predictions at 6 years, several aspects must be considered to assess their quality. We illustrate for 6 individuals in Fig. 4 the correlation between the predicted and true methylation values for the 91 CpGs contained in PedBE's signature. The correlations between predicted and observed methylation are high (mean Pearson = 0.99; mean Spearman = 0.98). We also checked the correlation of predicted and observed methylation values for each CpG (Figure S1), as well as the global prediction correlations for all CpGs (Figure S2).

The most compelling advantage of GP-based methods comes from the uncertainty quantification associated with the predictions. To underline this valuable feature, we provided a visualisation of errors, computed as the difference between predicted and observed values for all individual CpGs (Figure S3). Majority (≈80%) of the errors remain within 5% range (±0.05), and about 95% of the CpG sites present less than 10% methylation differences. In addition, we can observe that the vast majority of errors were expected and adequately quantified by the algorithm as we anticipated that 95% of the data points would fall within the CIs. The ratios of empirical errors contained within the 95% CI are, on average, 92.99% for skin&blood and 91.90% for PedBE, close to the theoretical value (Table 3).

**Table 3:** Performance metrics for methylation values predictions at age 6 years using different methods.

Epigenetic clock	# CpGs	Multi mean GPs		Individual/CpG mean		Mixed linear	48 month values
		RMSE	*CIC*_*95*_	RMSE	RMSE	RMSE	RMSE
PedBE	91	**0.046 (0.086)**	91.90 (27.30)	0.068 (0.112)	0.062 (0.104)	0.089 (0.122)	0.064 (0.099)
Skin&blood	368	**0.043 (0.068)**	92.99 (26.90)	0.115 (0.151)	0.089 (0.116)	0.072 (0.117)	0.063 (0.120)

We reported the mean (standard deviation) of the evaluated root mean squared error (RMSE) for multi-mean GPs and against several baselines. The 95% credible interval coverage (CIC95) is a calibration measure for uncertainty quantification (the closer to 95% the better).

Bold indicates the lowest RMSE across methods.

Multi-mean GP utilises a combination of both the mean trend at 6 years and the individual-specific pattern from prior measurements. This results in enhanced prediction accuracy and a reduced overall Root Mean Squared Error (Table 3). Although we mainly focused in the paper on the task of forecasting two years in the future, we further demonstrated the robustness and performance of multi-mean GPs for long-term forecasting. Instead of predicting only methylation at 72 months based on 3, 9 and 48-month data for each individual-CpG time series, we provided additional results when using 3 and 9-month data to forecast methylation values both at 48 and 72 months (Table S1, Figure S4). Prediction error of the multi-mean GP remains low even when forecasting several years into the future, with an uncertainty quantification that adjusts to the reduced available information from observed data.

### Epigenetic age can be estimated from predicted methylation profile

In addition to forecasting CpG status, considerable utility can be derived from investigating the relationship between methylation profiles and health outcomes from an individual. We explored how the DNA methylation time series can be leveraged to anticipate the evolution of biological outcomes, even in the long term, while propagating the uncertainty associated with our predictions. Here, we exemplify this by looking at the prediction of epigenetic age.

**Fig. 5:**
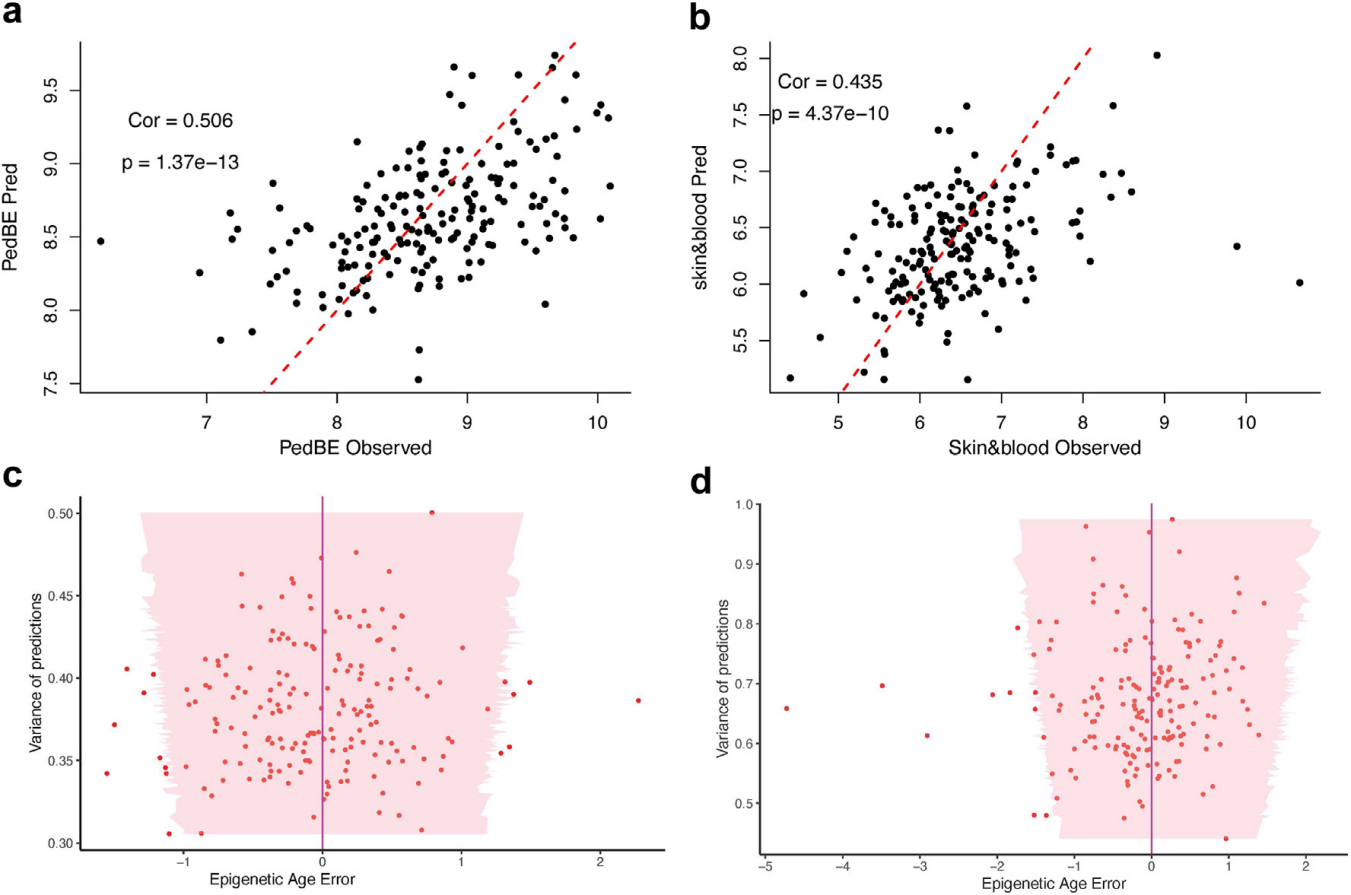
**Epigenetic age computed from predicted and observed methylation value and the variance of predictions**. Taking all the 188 subjects in the testing set, epigenetic ages computed from predicted methylation values are plotted against epigenetic age computed from observed methylation values for PedBE clock (**a**) and skin&blood clock (**b**). Each dot represents a subject, and Pearson correlation is used to compare observed and predicted values. The variance of the epigenetic age prediction (uncertainty quantification of the epigenetic age predictions) is plotted against errors (difference in epigenetic age from using predicted methylation and observed methylation value) for 188 individuals for PedBE(**c**) and skin&blood (**d**) respectively. For each individual (sorted by increasing uncertainty on the y-axis), the predicted mean ages using PedBE and skin&blood clocks on predicted methylation values at 6 years are used as a reference and displayed as a purple line; the pink region corresponds to the associated 95% credible intervals; each red dot corresponds to the epigenetic age computed using true observed methylation values.

The epigenetic age is computed from methylation values following skin&blood^21^ and PedBE^9^ clocks. An example of these estimations is depicted in Figure S5 for an individual. Our predictions are displayed as probability distributions, and we can see they accurately recover the true epigenetic age (red line) while accounting for the uncertainty propagated from the underlying methylation forecasts. We observed that the epigenetic age computed following the skin&blood clock is generally closer to the chronological age than with the PedBE clock. Epigenetic age estimated from PedBE is higher than chronological age for majority of the samples (Table S1, Figure S6). This trend has previously been observed in other studies.^23^^,^^24^ However, the epigenetic age computed from predicted methylation values and observed methylation values are well correlated for both PedBE and skin&blood (Fig. 5a and b). The Pearson correlation of epigenetic age calculated using predicted methylation value and observed methylation value is 0.506 (p = 1.37e-13) and 0.435 (p = 4.37e-10) for PedBE and skin&blood respectively. We have also shown the pairwise correlation of the epigenetic age across time points as well as the correlation obtained using the observed and predicted methylation values (Figures S7a, S8a).

We evaluated the predictive performance by displaying absolute errors with the associated uncertainty of the epigenetic age predictions for the 188 testing individuals (Fig. 5c and d). Similar to the methylation time series predictions, we remark that the range of empirical errors of the epigenetic age (red points) remains adequately recovered by the pink credible intervals. The mean accuracy between epigenetic age from predicted methylation value and observed methylation value range from 0.36 to 0.70 (Table S1). The multi-mean GPs approach proposed is able to achieve a credible interval coverage that remains close to the theoretical value of 95%. This property is especially reassuring from a practical point of view as it indicates that high uncertainty should be dealt with additional caution when conducting inference.

### Predicted age acceleration is associated with child health outcomes

**Fig. 6:**
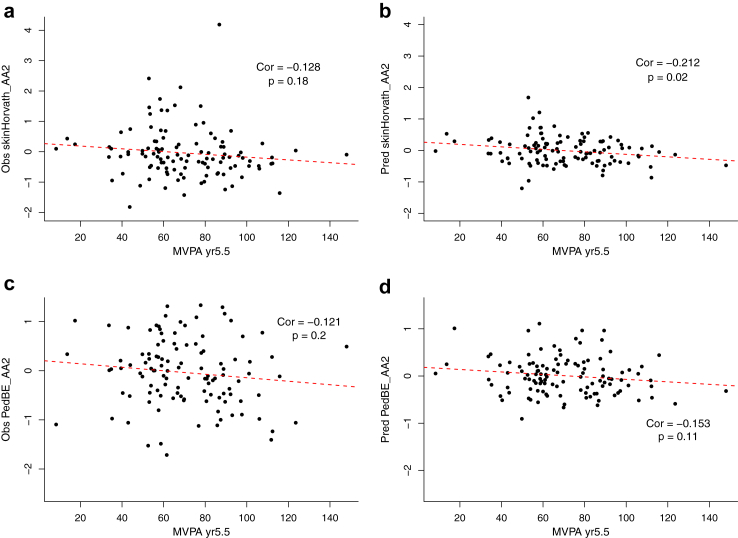
**Scatter plot of age acceleration plotted against MVPA measured at age 5.5 years**. Age acceleration computed using skin&blood clock from observed and predicted methylation value (**a, b**) and PedBE observed and predicted methylation value (**c, d**) plotted against moderate to vigorous activity (MVPA). Each dot represents a subject in the testing set. The red dotted line is the regression line, and Pearson correlations are reported.

Using the epigenetic age computed from the predicted methylation values, we have also computed the AA, defined as the residuals of regressing epigenetic age and chronological age^7^ for the participants. We have shown the pairwise correlation of the AA of PedBE and skin&blood (Figures S7b, S8b). The correlation between AA computed from observed and predicted methylation value is 0.51 and 0.43 respectively for the PedBE and skin&blood. This is similar to the correlation observed in the epigenetic age computed using predicted and observed methylation values. We then performed association tests with clinical variables such as moderate to vigorous physical activity (MVPA) and measured blood pressure. The results obtained from both observed and predicted methylation values are similar, though the significant levels are different (Fig. 6).

We notice that higher MVPA at age 5.5 years old is correlated with lower AA for skin&blood from observed (Pearson corr = −0.128) and predicted (Pearson corr = −0.212) methylation profiles. The directionality observed is consistent across AA computed from observed and predicted methylation values in both skin&blood and PedBE clocks. In addition, we also observed higher diastolic blood pressure is correlated with higher AA calculated from predicted methylation values at 6 years (PedBE: Corr_predicted_ = 0.171, skin&blood: Corr_predicted_ = 0.182). Although these subjects did not present significant associations between measured AA and systolic blood pressure, it remains noteworthy that both associations present similar trends (Figure S9). Overall, the results from predicted methylation values appear as a faithful reflection of methylation status from early time points, which always constitutes valuable guidance for practitioners.

## Discussion

With a probabilistic machine learning approach, we are able to forecast the methylation profile of an individual 2 years into future. Applying the proposed multi-mean GP model to the methylation longitudinal data achieved high accuracy estimation of epigenetic AA and MVPA from the predicted methylation values. Higher MVPA is consistently associated with lower AA in school-going children. A recent study has also reported a similar correlation for adults.^25^ It is particularly reassuring to observe the consistency of these trends in MVPA and age acceleration across different age groups and different samples.

Currently, we have limited the models to a subset of CpGs required to compute specific epigenetic age. Nevertheless, our algorithm can be scaled to make predictions for all potential methylation sites in the human genome, since computational complexity increases linearly with the number of individuals and CpGs. This would enable the identification and validation of epigenetic signatures associated with multiple health conditions, such as obesity^26^^,^^27^ and mental health.^12^ Such advances would contribute vastly to the research community conducting cross-sectional and longitudinally epigenetic studies.

We were able to make accurate predictions of methylation status around age six based on methylation profiles collected from three early time points (3, 9, and 48 months). There is potential for our method to make predictions with greater certainty if we trained on data recorded more often during the period of measures. Nonetheless, the proposed algorithm can easily include more densely sampled data to train mean processes on a broader range of ages, resulting in more accurate predictions. Optimising the number of data points needed to forecast the methylation profile X years into the future will help researchers plan their studies and determine when to collect data from their subjects. Potential future studies could assess epigenetic ageing processes throughout the whole lifespan of an individual, from infancy to elderly, without incurring additional costs or burdens for the participant to donate more samples.

One limitation with this study is the difficulty in replicating in a similar large-scale longitudinal dataset, such as a dataset with at least 4 timepoints from the same platform to show the generalisability of our model. Table S2 shows the existing longitudinal methylation datasets from population cohorts with three or more timepoints. As seen in the table, only our cohort dataset (GUSTO) and Lothian Birth Cohort meet these criteria. The Lothian Birth Cohort was not suitable for this study because its fourth timepoint was profiled using an array (EPIC850K) that is different to the one used in previous timepoints (Inf450k). We also acknowledge that we are lacking a ground-truth for biological ages, however, this is a common limitation with all other epigenetic clock studies. We assumed that lifestyle and environmental factors have minimal influence on our subjects at a young age, and thus, their biological age is very close to their chronological age. However, we note that current epigenetic clock estimates of biological age may be inaccurate if substantial lifestyle or environmental changes occur between the observed and predicted time points.

From a methodological perspective, GPs are known for their computational limitations when the number of time points grows. Although this aspect is less of a concern in this study as we only collected four time points per time series (since measuring methylation remains costly), different applications of multi-mean GPs could fall into bottlenecks when modelling high-frequency time series. On the other hand, despite its remarkable linear complexity in the number of individuals and CpGs, meaning that modelling all individual-CpG time series altogether is roughly as costly as modelling them independently, our approach could still benefit from significant improvements in terms of scaling by leveraging clever approximations, such as stochastic EM for instance. In this paper, we used clocks based on a few hundred CpGs, allowing us to perform all computations on a simple laptop in a few hours without parallelisation. However, more sophisticated problems could involve thousands, or millions of correlated time series, and such contexts would require implementing additional techniques to improve scalability and expand the range of potential applications, which constitutes an exciting avenue for future research. Finally, we acknowledge that extrapolation is especially challenging for long-term time series, and such GP-based predictions will typically converge, with increasing uncertainty, towards the prior distribution as we move away from observed data.^19^

In conclusion, we present an attempt to predict DNA methylation profiles of human individuals multiple years into the future. Our machine learning approach facilitates longitudinal studies of development, ageing, and disease progression using molecular data from a limited number of time points. This algorithm not only predicts future data by leveraging available longitudinal data but also generates predictions for any time point, enabling interpolation and imputation of missing data. Additionally, we have demonstrated that the uncertainty associated with these predictions is well-calibrated, providing downstream users with insight into the confidence they should place in each forecast.

## Contributors

Conceptualisation of study by DW, AL, ALT, MAA. Data processing was carried out by ALT. Analyses and manuscript preparation was performed by AL and ALT. ALT and AL have accessed and verified underlying data used. Review and editing was carried out by DW, FD, MAA. All authors read and approved the final version of manuscript.

We want to thank all GUSTO participants who contributed samples and the GUSTO study group.

The GUSTO study group includes: Airu Chia, Andrea Cremaschi, Anna Magdalena Fogel, Anne Eng Neo Goh, Anne Rifkin-Graboi, Anqi Qiu, Arijit Biswas, Bee Wah Lee, Birit Froukje Philipp Broekman, Candida Vaz, Chai Kiat Chng, Chan Shi Yu, Choon Looi Bong, Daniel Yam Thiam Goh, Dawn Xin Ping Koh, Dennis Wang, Desiree Y. Phua, E Shyong Tai, Elaine Kwang Hsia Tham, Elaine Phaik Ling Quah, Elizabeth Huiwen Tham, Evelyn Chung Ning Law, Evelyn Keet Wai Lau, Evelyn Xiu Ling Loo, Fabian Kok Peng Yap, Falk Müller-Riemenschneider, Franzolini Beatrice, George Seow Heong Yeo, Gerard Chung Siew Keong, Hannah Ee Juen Yong, Helen Yu Chen, Hong Pan, Huang Jian, Huang Pei, Hugo P S van Bever, Hui Min Tan, Iliana Magiati, Inez Bik Yun Wong, Ives Lim Yubin, Ivy Yee-Man Lau, Jacqueline Chin Siew Roong, Jadegoud Yaligar, Jerry Kok Yen Chan, Jia Xu, Johan Gunnar Eriksson, Jonathan Tze Liang Choo, Jonathan Y. Bernard, Jonathan Yinhao Huang, Joshua J. Gooley, Jun Shi Lai, Karen Mei Ling Tan, Keith M. Godfrey, Keri McCrickerd, Kok Hian Tan, Kothandaraman Narasimhan, Krishnamoorthy Naiduvaje, Kuan Jin Lee, Li Chen, Lieng Hsi Ling, Lin Lin Su, Ling-Wei Chen, Lourdes Mary Daniel, Lynette Pei-Chi Shek, Maria De Iorio, Marielle V. Fortier, Mary Foong-Fong Chong, Mary Wlodek, Mei Chien Chua, Melvin Khee-Shing Leow, Michael J. Meaney, Michelle Zhi Ling Kee, Min Gong, Mya Thway Tint, Navin Michael, Neerja Karnani, Ngee Lek, Noor Hidayatul Aini Bte Suaini, Oon Hoe Teoh, Peter David Gluckman, Priti Mishra, Queenie Ling Jun Li, Sambasivam Sendhil Velan, Seang Mei Saw, See Ling Loy, Seng Bin Ang, Shang Chee Chong, Shiao-Yng Chan, Shirong Cai, Shu-E Soh, Stephen Chin-Ying Hsu, Suresh Anand Sadananthan, Swee Chye Quek, Tan Ai Peng, Varsha Gupta, Victor Samuel Rajadurai, Wee Meng Han, Wei Wei Pang, Yap Seng Chong, Yin Bun Cheung, Yiong Huak Chan, Yung Seng Lee, Zhang Han.

## Data sharing statement

Our source code is available at public repository https://github.com/ArthurLeroy/MultiMeanGP. Raw methylation data has been deposited to NCBI GEO accession number GSE254135. The clinical data may be requested via GUSTO data portal: https://gustodatavault.sg/.

## Declaration of interests

FD is an employee of Novartis Biomedical Research and a shareholder of Novartis and Roche. All other authors report no financial relationships with commercial interests.

## Appendix A Supplementary data

Supplementary-revised
