# Supplementary material for: Longitudinal prediction of DNA methylation to forecast epigenetic outcomes
Source: eBioMedicine. 2025 Apr 22;115:105709. doi: 10.1016/j.ebiom.2025.105709 (PMC12051112; doi:10.1016/j.ebiom.2025.105709)
Supplement: Supplementary-revised [file mmc1.docx]

**Supplementary Materials**


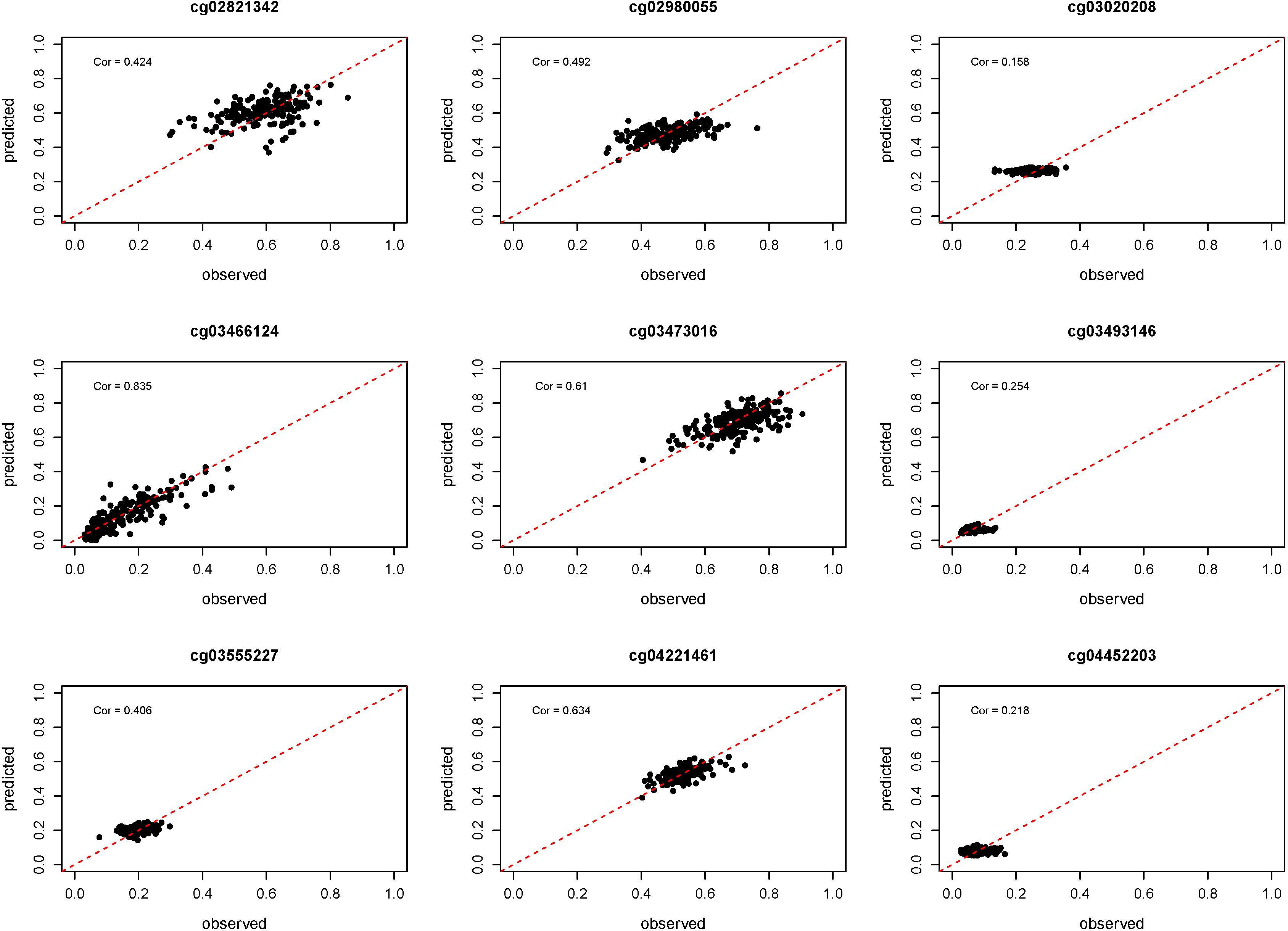


**Figure** **S1** Example of predicted methylation values (y-axis) plotted against observed methylation values (x-axis) for the CpGs in PedBE clock. Each dot represents an individual and the red dotted line is the x=y line.


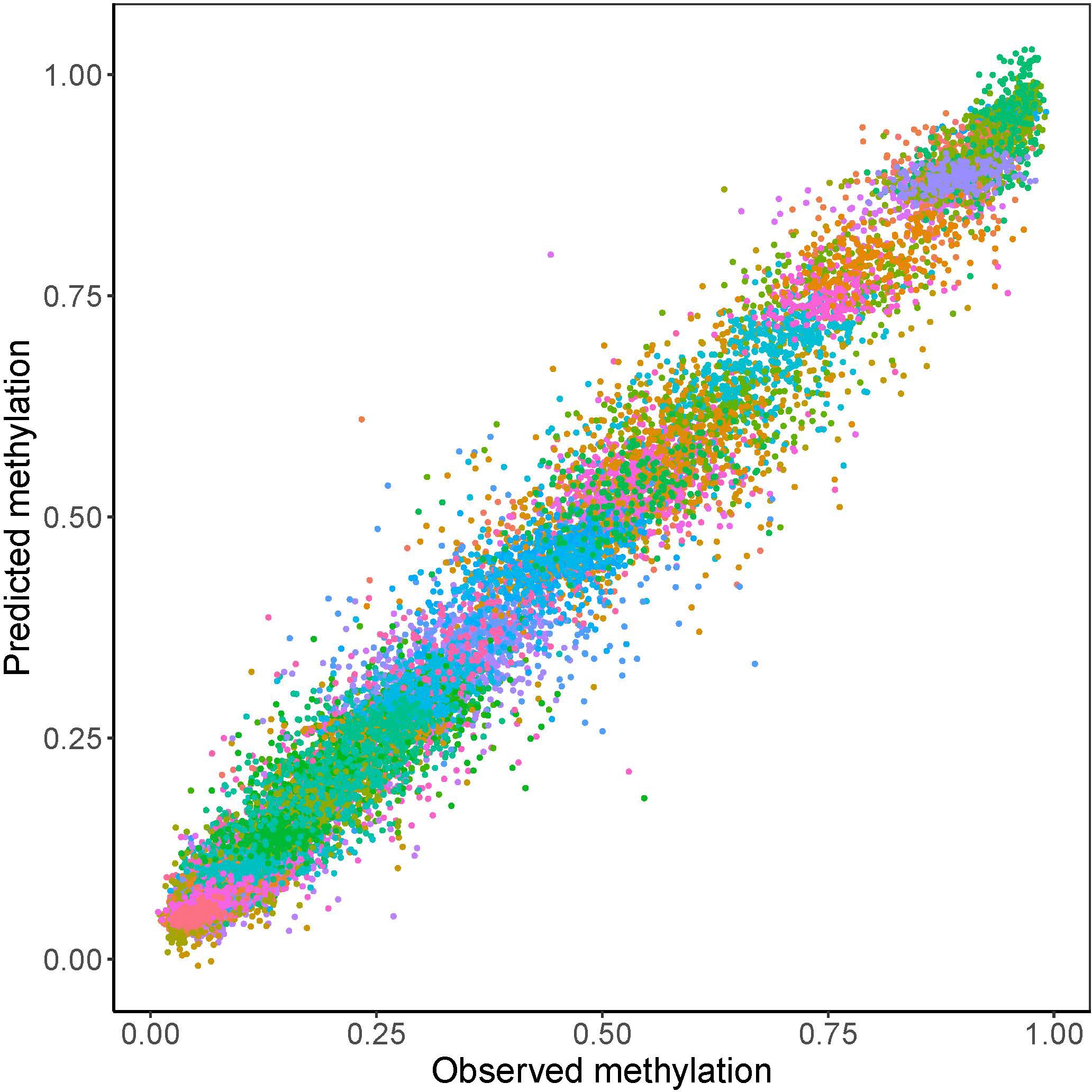


a


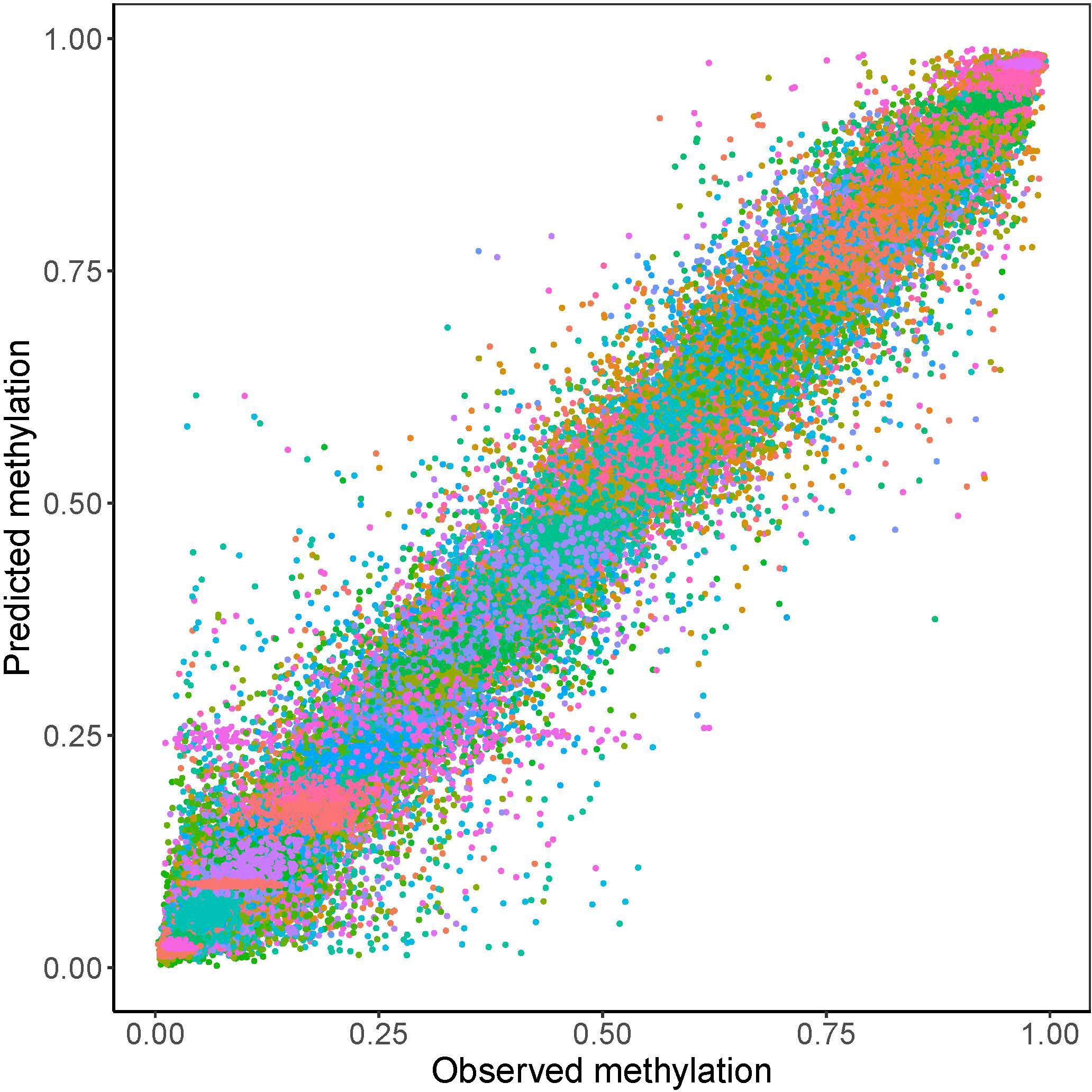


b

**Figure S2** Mean predicted values (y-axis) plotted against observed methylation values (x-axis) for each CpG-individual couple of the testing set (188 individuals) involved in the PedBE clock (91 CpGs, a) and the Horvath skin&blood clock (368 CpGs, b). Each colour corresponds to a specific CpG.


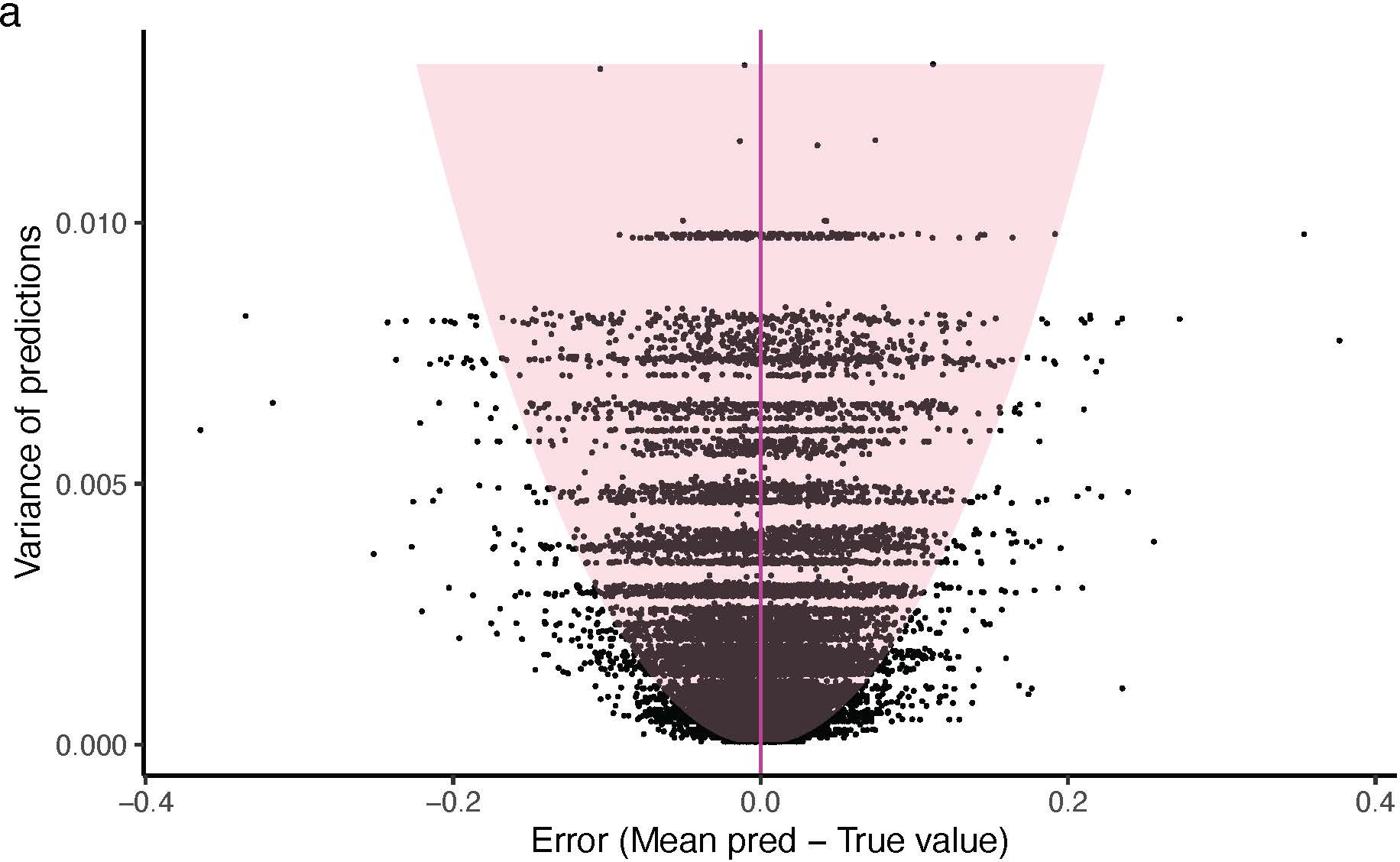

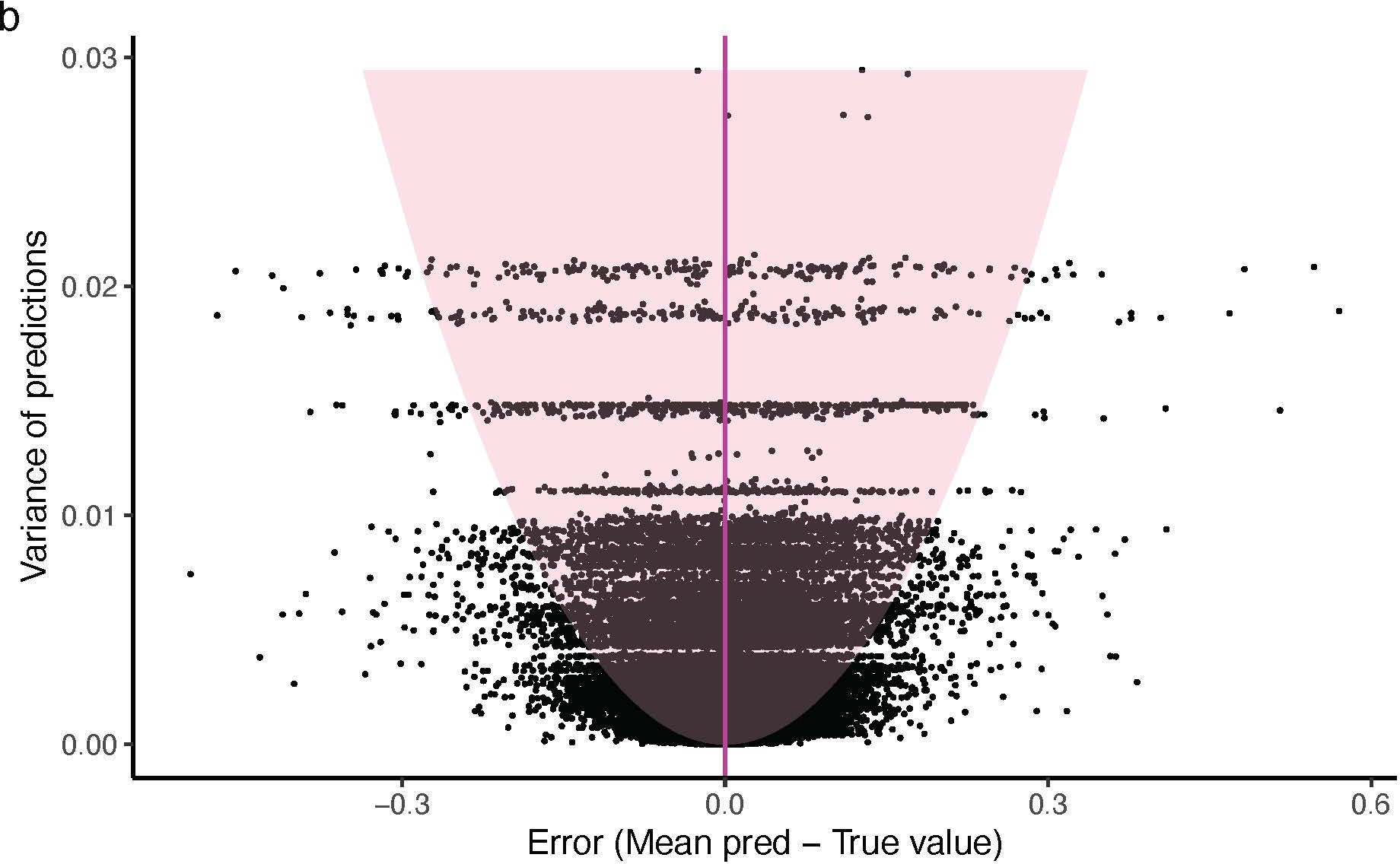


**Figure S3** Distribution of prediction errors (differences between predicted and observed methylation value) sorted by predictive variance for both PedBE clock (a) and skin&blood (b). For each individual-CpG couple of the testing set (188 individuals), each error value is displayed as a black dot, and the pink region represents the 95% credible interval associated with the predicted mean (purple vertical line).


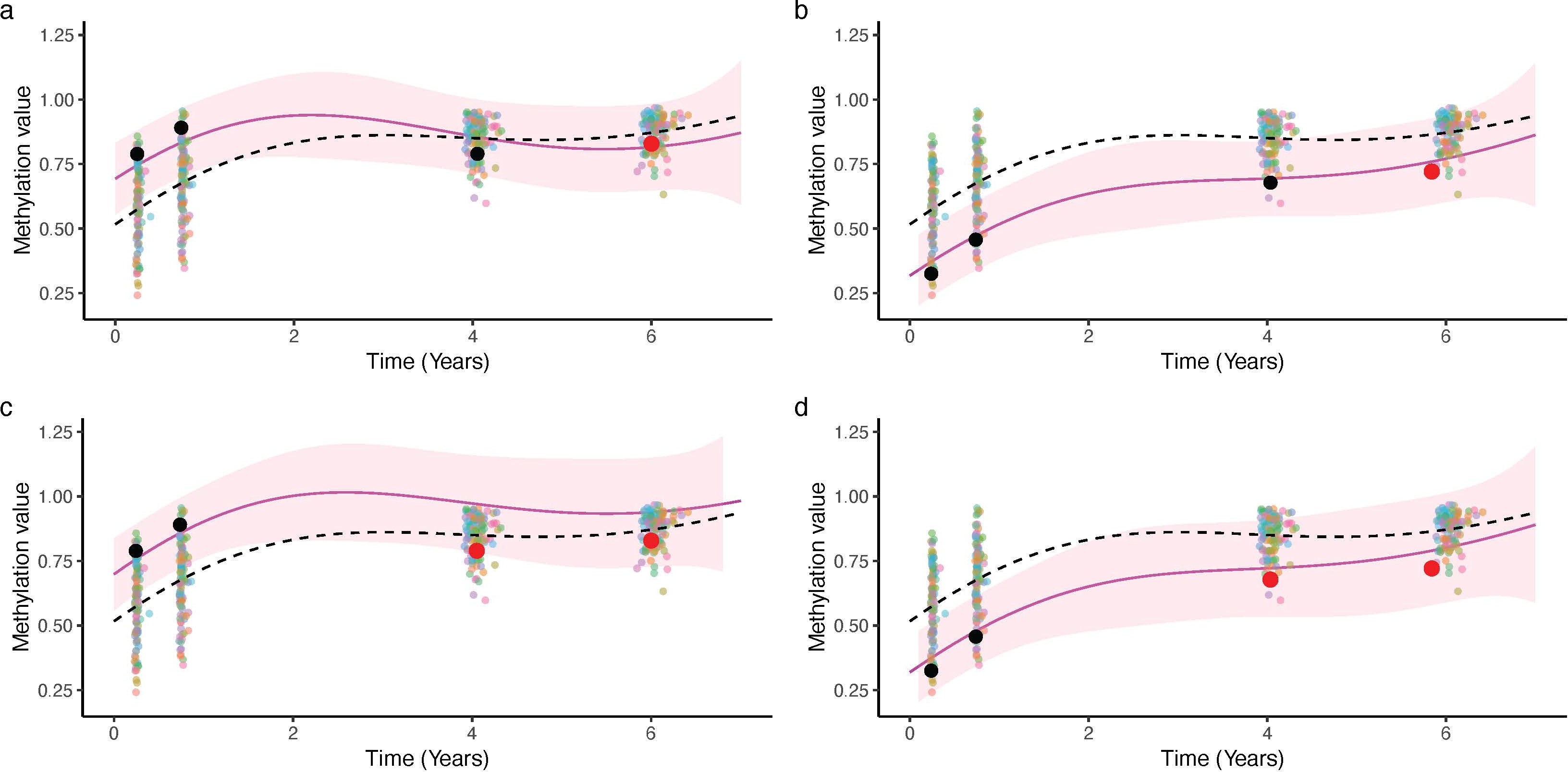


**Figure S4** CpG-specific mean processes and individual-specific predictions. Multi-mean GPs prediction curve

(pink) with associated 95% credible intervals (pink band), and comparison with observed values at 6 years (a and b) and at 4 and 6 years (c and d) for two illustrative individuals. Observed points are coloured in black, while the testing points are in red. Background points correspond to the training observations coloured by individuals. The dashed line represents the mean curve from the CpG-specific mean process.


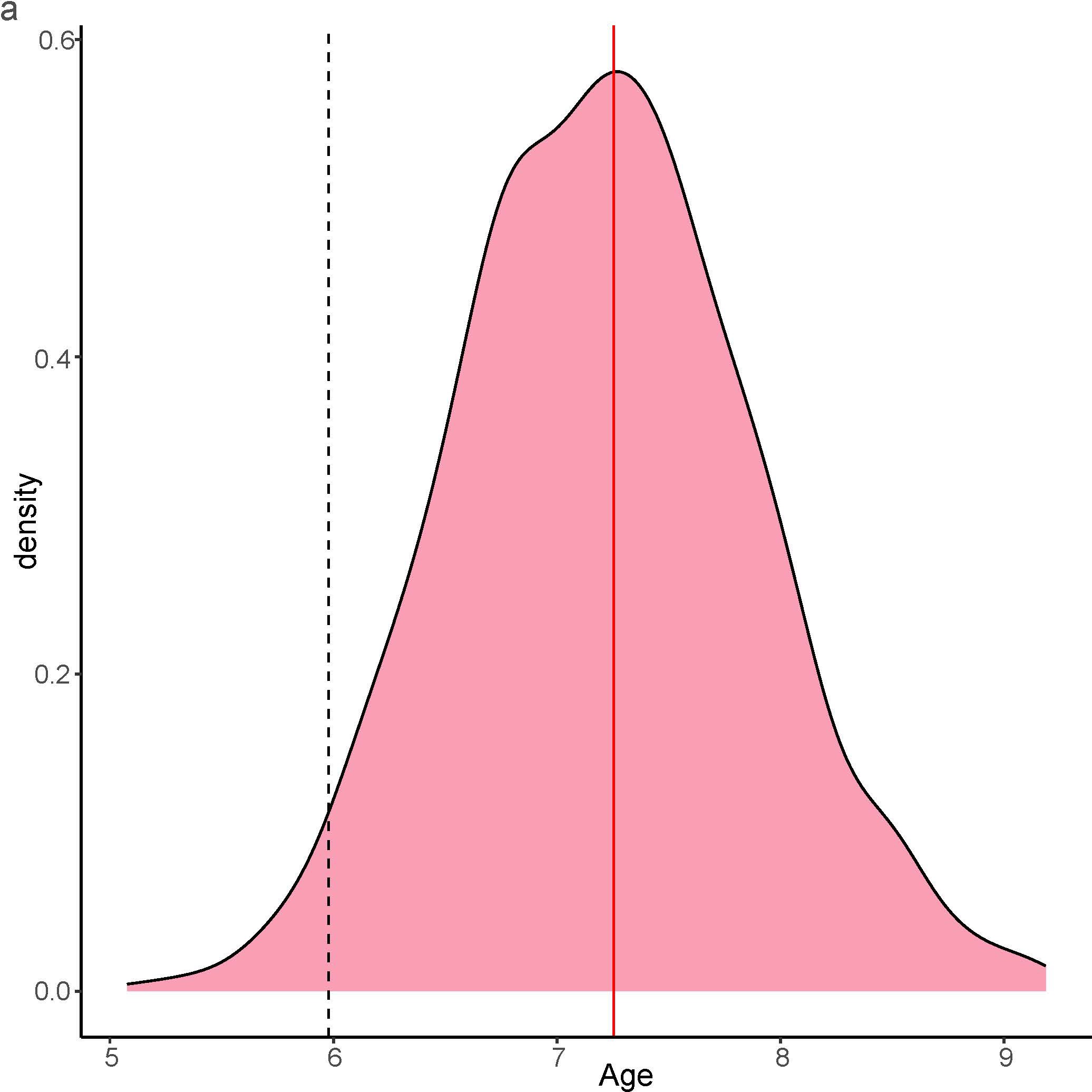

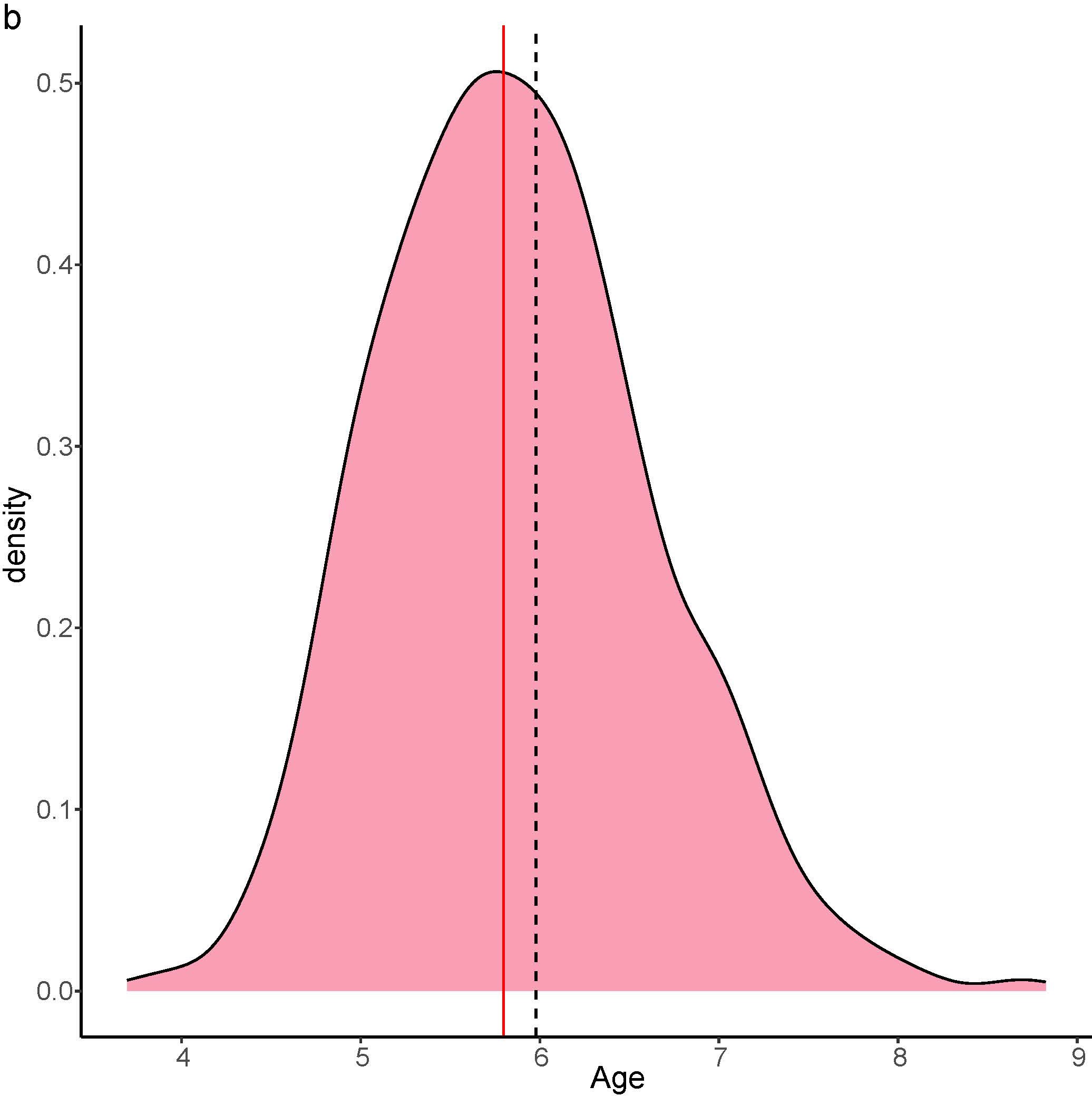


**Figure S5** Posterior distribution of the epigenetic age (pink area) estimated from PedBE (**a**) and Horvath skin and blood (**b**) clocks, using *CpG predictions* at 6 years for the same illustrative individual. The true age is displayed as a dashed black line, while the red vertical line represents the epigenetic age estimated from the *true CpG values* at 6 years.


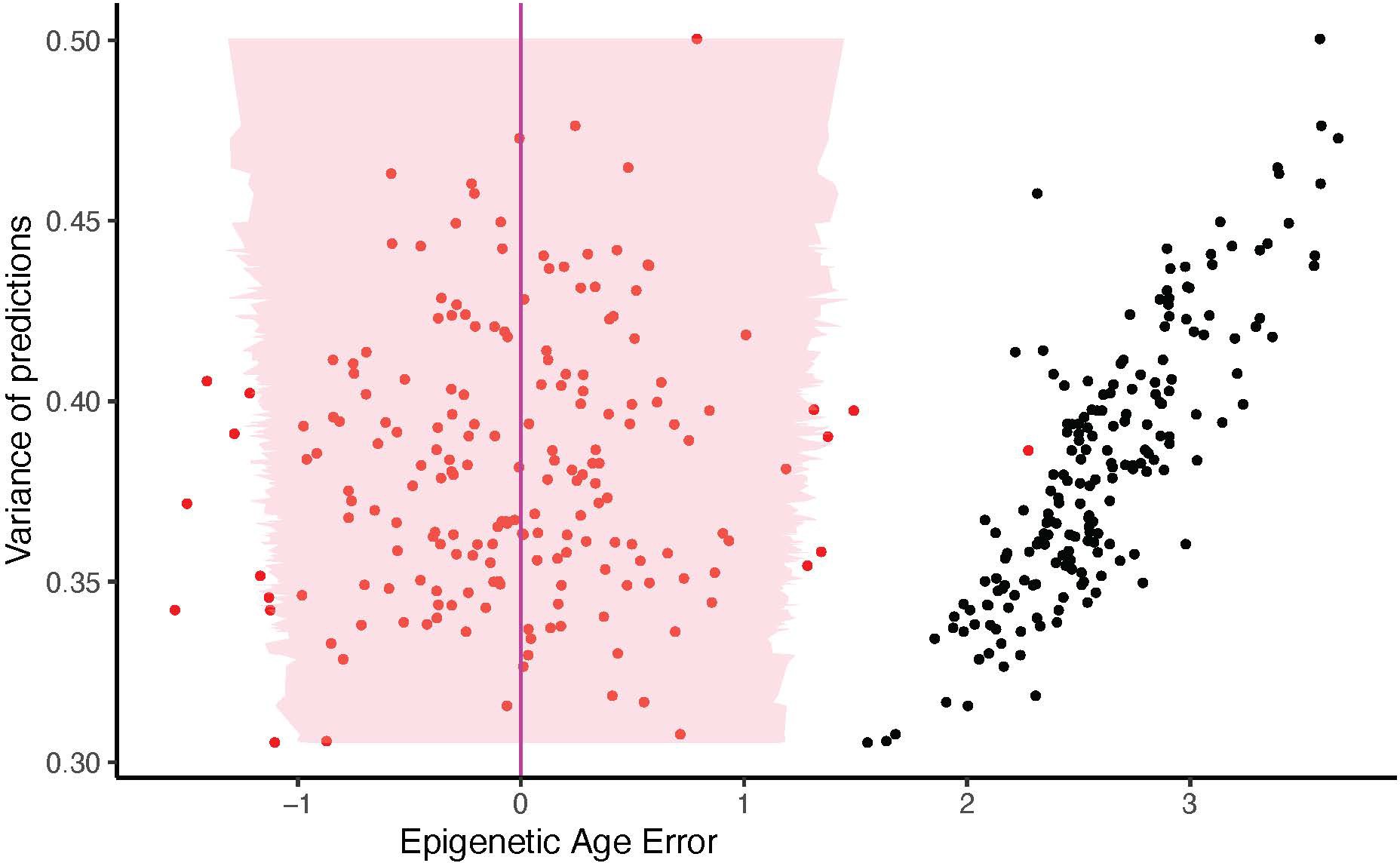
a


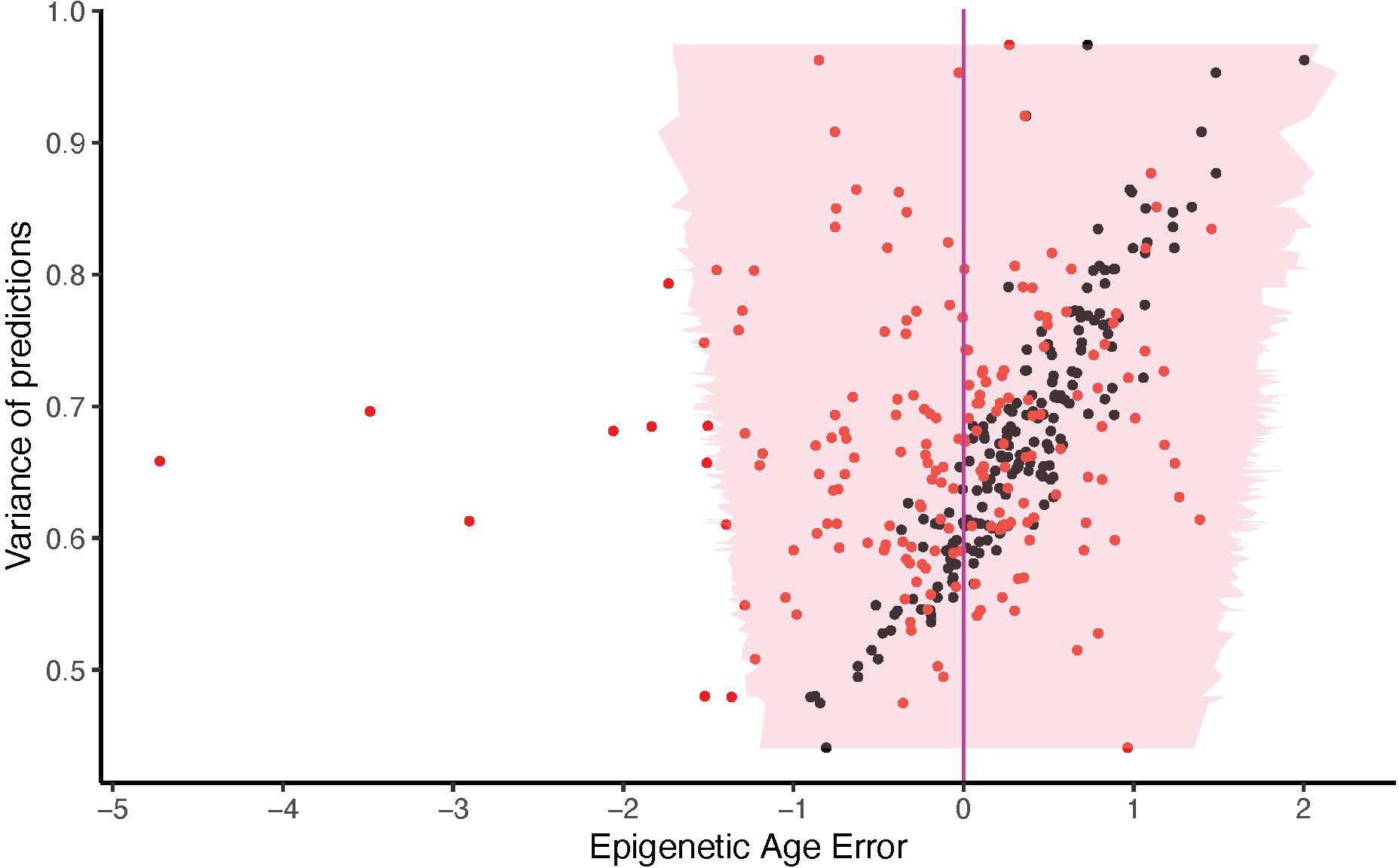


b

**Figure S6** Illustration of the bias between epigenetic age and chronological age for 188 testing individuals when estimated from the PedBE clock (**a**) compared with the Horvath skin&blood clock (**b**). Our epigenetic age predictions are used as reference (x=0) and displayed as a purple vertical line; the pink region corresponds to the associated 95% credible intervals; each red dot corresponds to the error between our predicted epigenetic age and epigenetic age computed from observed *methylation* values at year 6. Each black dot corresponds to the error between our predicted epigenetic age and observed chronological age.


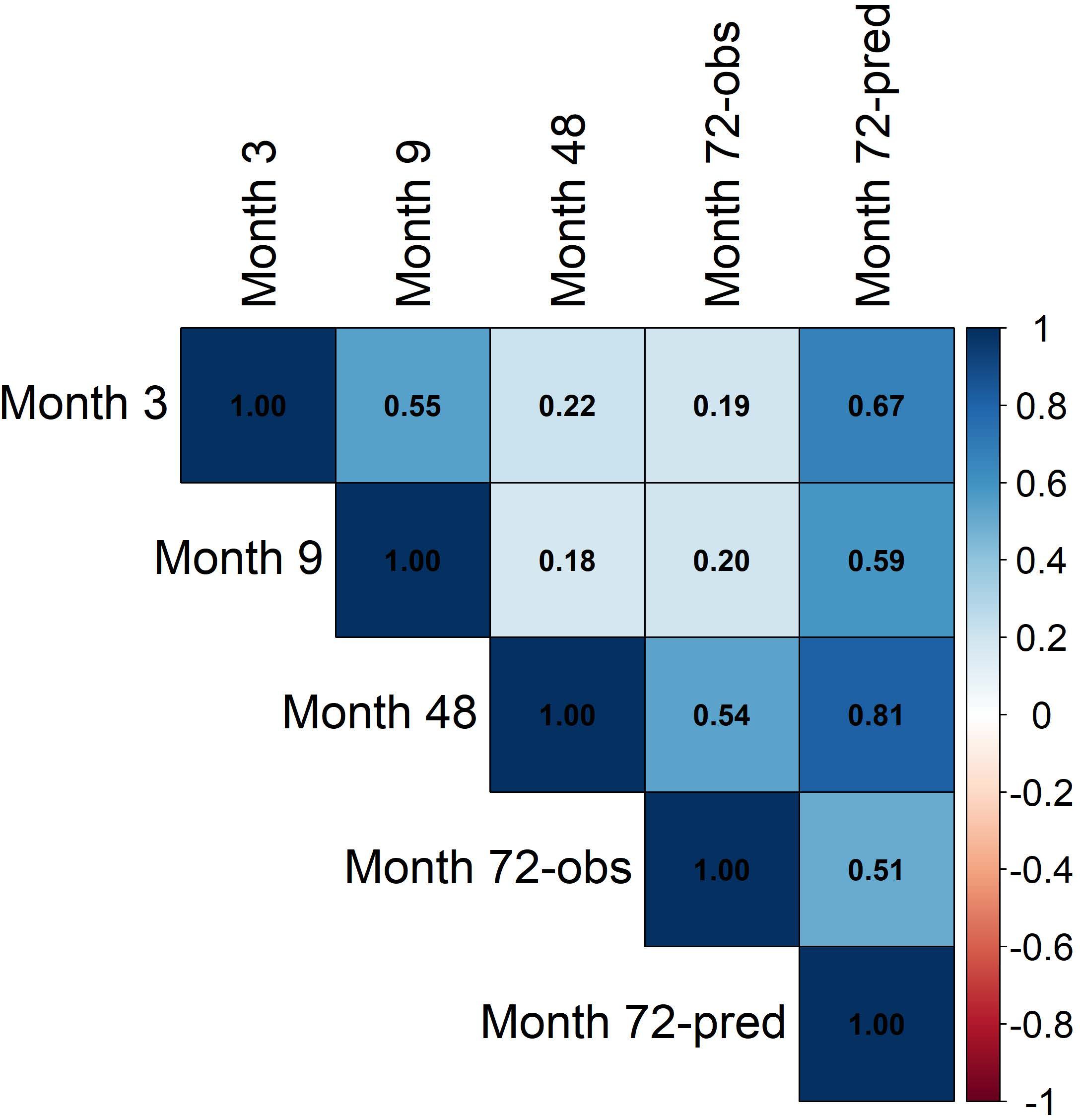
a PedBE

# PedBE - AA

b


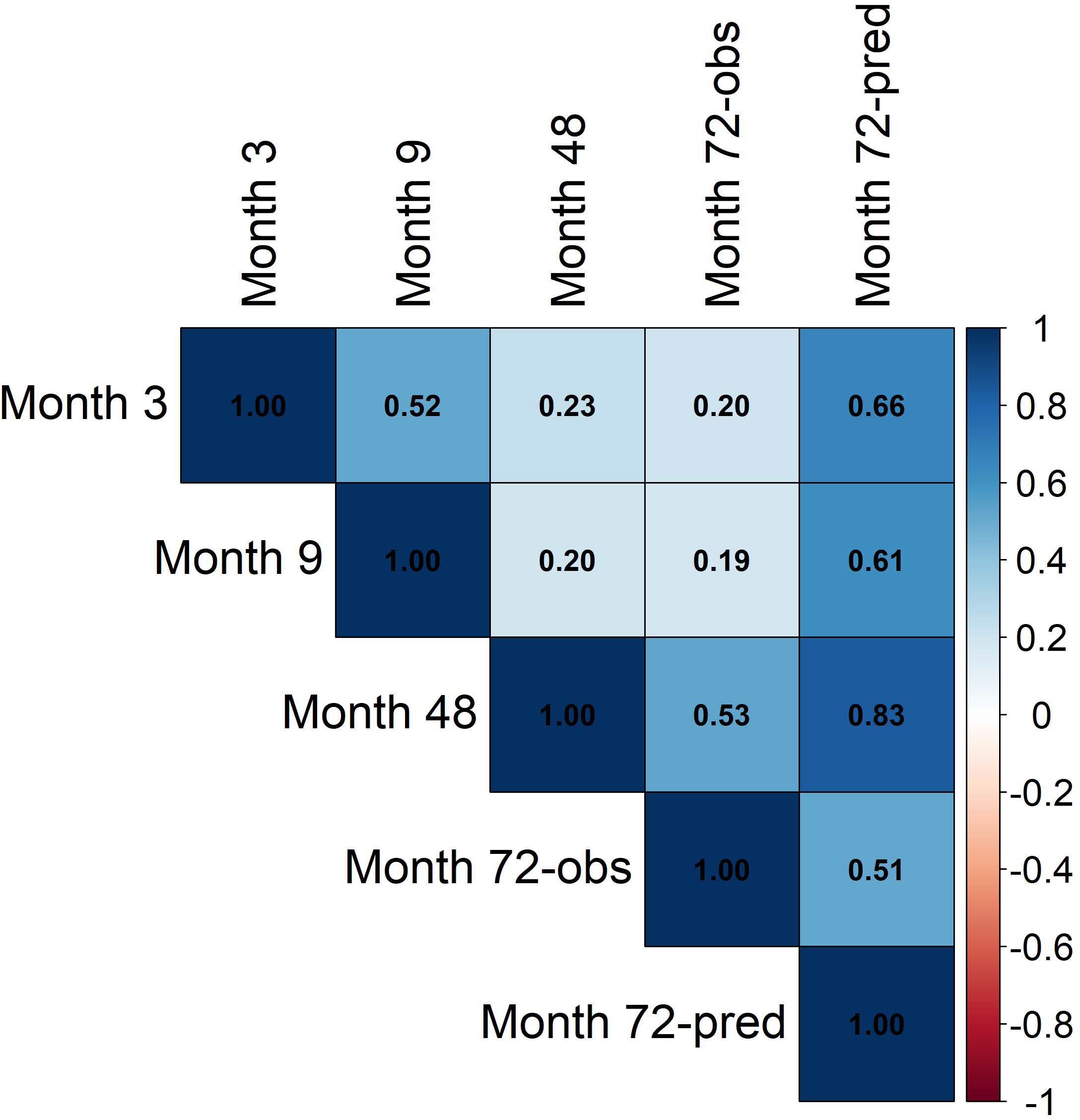


**Figure S7** Correlation across different timepoints between epigenetic age (a) computed using PedBE clock and age acceleration (b). There are two sets of data for Month 72, one computed using the observed methylation value (Month 72- obs) and one computed using the predicted methylation value (Month 72 -pred). Age acceleration is defined as the residual of regressing chronological age onto epigenetic age.

a

#
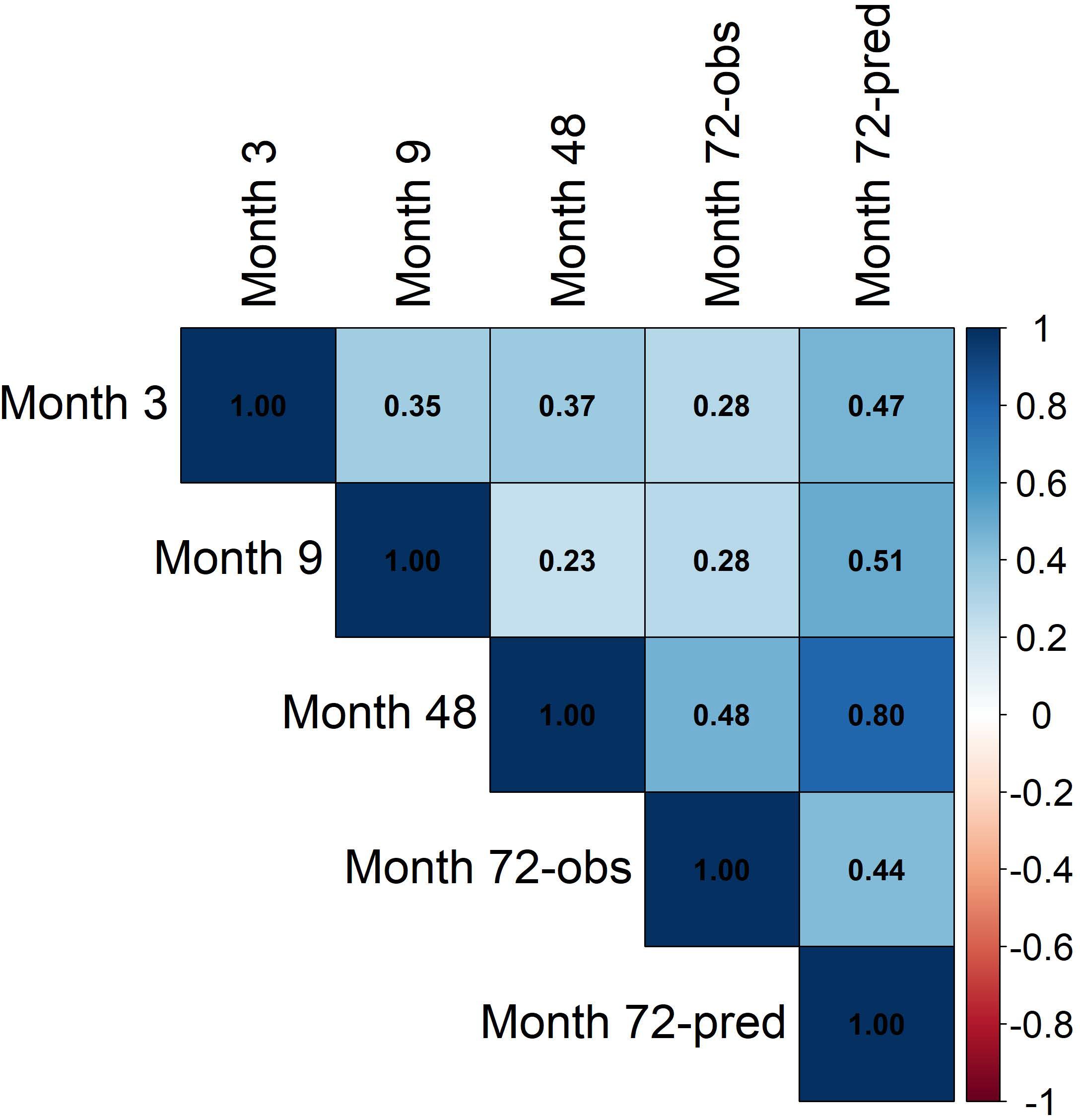
skin&blood

b skin&blood - AA


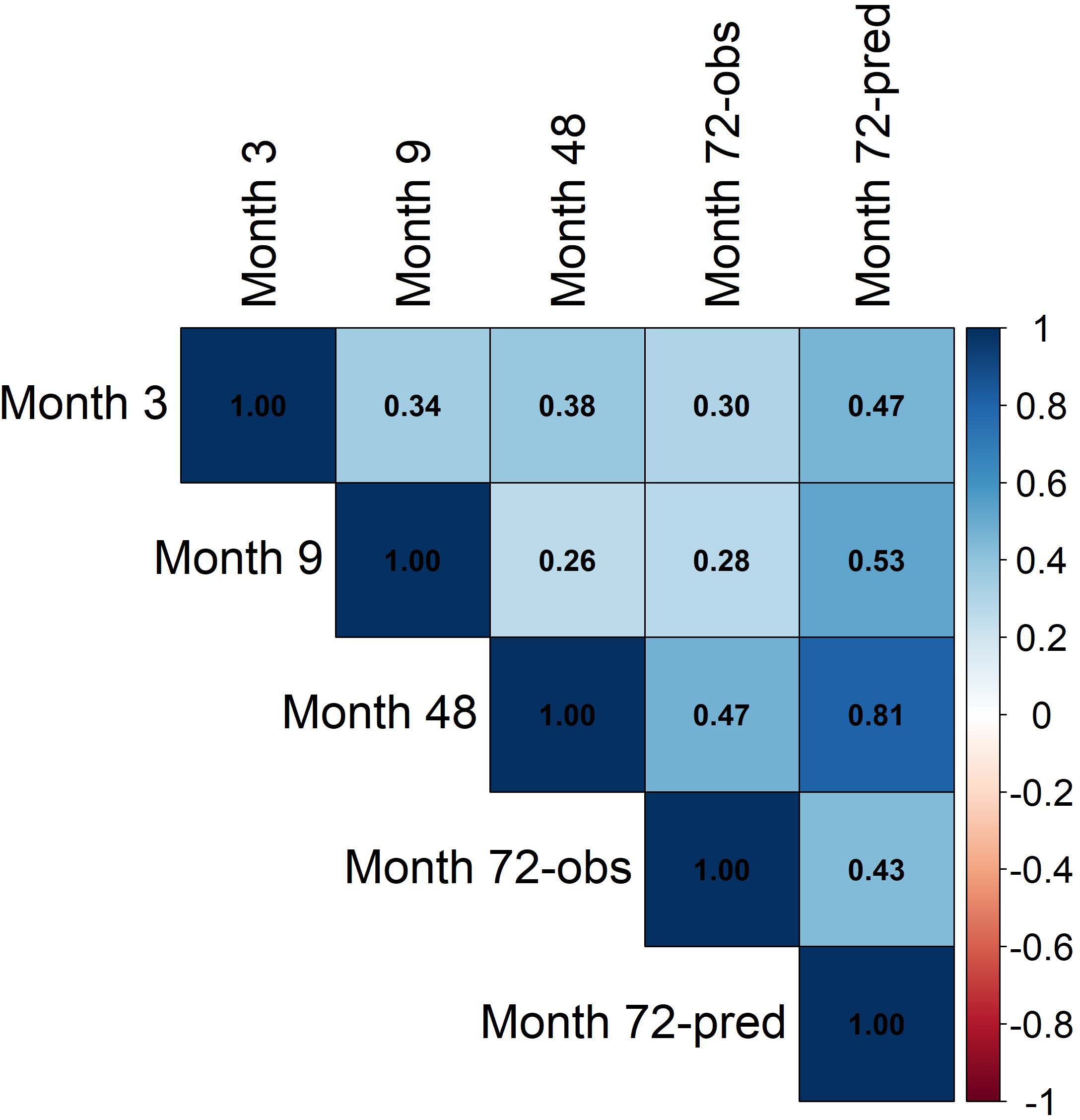
**Figure S8** Correlation across different timepoints between epigenetic age (a) computed using skin&blood clock and age acceleration (b). There are two sets of data for Month 72, one computed using the observed methylation value (Month 72- obs) and one computed using the predicted methylation value (Month 72 -pred). Age acceleration is defined as the residual of regressing chronological age onto epigenetic age.


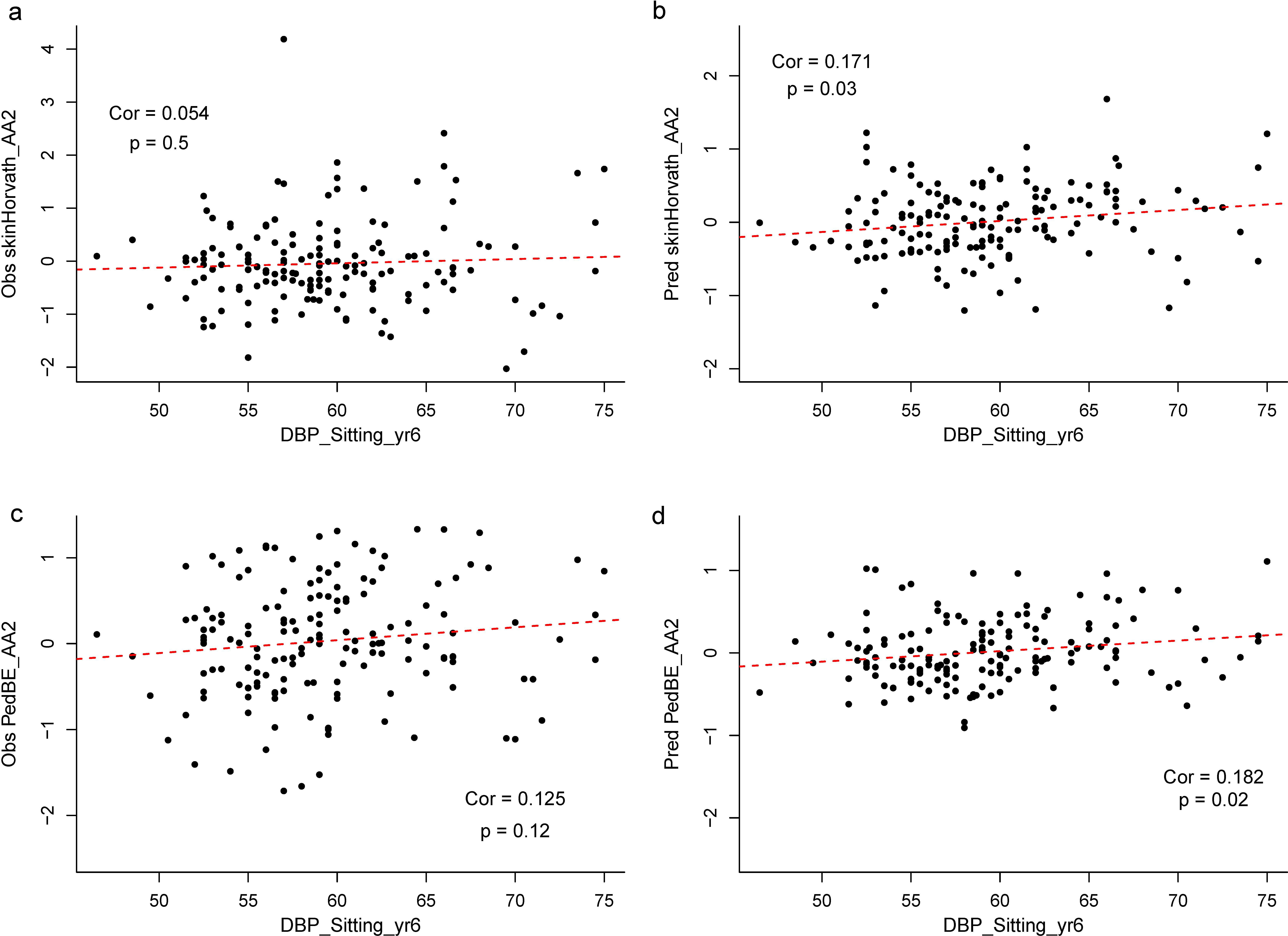


**Figure S9** Scatter plot of age acceleration against diastolic blood pressure measured at 6 years old.

**Table S1** Average (sd) values of RMSE and *CIC*_95_ of Multi-mean GPs predictions for 188 testing individuals, using Horvath skin&blood (368 CpGs) and PedBE (91 CpGs) clocks. Experiments have been conducted on two settings, using 3, 9, 48-month data points to forecast 72-month methylation values and using 3, 9-month data points to forecast 48 and, 72-month methylation values.

| **Epigenetic Clock** | **# CpGs** | **Prediction at 72 months** | | **Prediction at 48 and 72 months** | |
| --- | --- | --- | --- | --- | --- |
|  |  | RMSE | *CIC_95_* | RMSE | *CIC_95_* |
| PedBE | 91 | 0.046 | 91.9 | 0.052 | 89.92 |
|  |  | (0.086) | (27.30) | (0.092) | (30.11) |
| Skin&blood | 368 | 0.043 | 92.99 | 0.049 | 89.98 |
|  |  | (0.068) | (26.90) | (0.090) | (30.03) |

**Table S2** Overview of the largest population cohorts with longitudinal DNA methylation data.

| **Cohort** | **Sample Size** | **Ages** | **Timepoints** | **Platform** | **Description** |
| --- | --- | --- | --- | --- | --- |
| InChianti | 699 | 21-95 | two follow-up or more timepoints (in the 1998, 2007, and 2013 study visits) | Inf450k | A total of 1721 samples from 699 participants (376 participants measured at two timepoints, 323 participants measured at three timepoints) were used in the analysis. |
| Lothian Birth Cohort (LBC1921) | 430 | ~79 yrs | wave 1 | Inf450k | Methylation data from blood sample are available in three waves in LBC1921 (wave 1, 3, and 4) and in four waves in LBC1936 (wave 1, 2, 3, and 4) |
|  | 173 | ~87 yrs | wave 3 | Inf450k |  |
|  | 82 | ~90 yrs | wave 4 | Inf450k |  |
| Lothian Birth Cohort (LBC1936) | 898 | ~ 70yrs | wave 1 | Inf450k | Methylation data from blood sample are available in three waves in LBC1921 (wave 1, 3, and 4) and in four waves in LBC1936 (wave 1, 2, 3, and 4) |
|  | 793 | ~ 73 yrs | wave 2 | Inf450k |  |
|  | 607 | ~76 yrs | wave 3 | Inf450k |  |
|  | 502 | ~79 yrs | wave 4 | EPIC850k |  |
| ALSPAC | 905 | birth | birth | Inf450k | cord blood sample |
|  | 969 | 7.5 yrs | timepoint 1 | Inf450k |  |
|  | 958 | 17 yrs | timepoint 2 | Inf450k |  |
| GUSTO | 203 | 3 mths | timepoint 1 | EPIC850k | Our data generated from buccal swab sample of a cohort of children born in 2010. |
|  | 323 | 9 mths | timepoint 2 | EPIC850k |  |
|  | 342 | 48 mths | timepoint 3 | EPIC850k |  |
|  | 800 | 72 mths | timepoint 4 | EPIC850k |  |
